# An annotated CNS transcriptome of the medicinal leech, *Hirudo verbana*: *De novo* sequencing to characterize genes associated with nervous system activity

**DOI:** 10.1371/journal.pone.0201206

**Published:** 2018-07-20

**Authors:** Adam J. Northcutt, Eva K. Fischer, Joshua G. Puhl, Karen A. Mesce, David J. Schulz

**Affiliations:** 1 Division of Biological Sciences, University of Missouri-Columbia, Columbia, Missouri, United States of America; 2 Department of Biology, Stanford University, Stanford, California, United States of America; 3 Department of Entomology and Graduate Program in Neuroscience, University of Minnesota, Saint Paul, Minnesota, United States of America; McGill University Department of Physiology, CANADA

## Abstract

The medicinal leech is one of the most venerated model systems for the study of fundamental nervous system principles, ranging from single-cell excitability to complex sensorimotor integration. Yet, molecular analyses have yet to be extensively applied to complement the rich history of electrophysiological study that this animal has received. Here, we generated the first *de novo* transcriptome assembly from the entire central nervous system of *Hirudo verbana*, with the goal of providing a molecular resource, as well as to lay the foundation for a comprehensive discovery of genes fundamentally important for neural function. Our assembly generated 107,704 contigs from over 900 million raw reads. Of these 107,704 contigs, 39,047 (36%) were annotated using NCBI’s validated RefSeq sequence database. From this annotated central nervous system transcriptome, we began the process of curating genes related to nervous system function by identifying and characterizing 126 unique ion channel, receptor, transporter, and enzyme contigs. Additionally, we generated sequence counts to estimate the relative abundance of each identified ion channel and receptor contig in the transcriptome through Kallisto mapping. This transcriptome will serve as a valuable community resource for studies investigating the molecular underpinnings of neural function in leech and provide a reference for comparative analyses.

## Introduction

Medicinal leeches have long been studied as a model for understanding the neural underpinnings of behavior [1–4], fundamental electrophysiology principles [5,6], central-pattern generation [7], and metamodulation [8]. Molecular sequence information for the Northwestern European medicinal leech *Hirudo medicinalis* has more recently been accumulating through studies targeting specific candidate gene sequences [9–14], tissue types [15,16], or life stages [17]. The Southeastern European medicinal leech *Hirudo verbana* has not received the same attention in the surge to bring transcriptomic approaches to traditionally “non-genetic” systems, even though it remains the most commonly used species in neurophysiological and behavioral studies [18–21]. While *H*. *medicinalis* and *H*. *verbana* have sometimes been confounded as a single species, they not only have distinct coloration and patterns, but also are genetically distinct, as shown through random amplified polymorphic DNA (RAPD) analyses [22] and mitochondrial genomic (mtDNA) sequencing [23]. While mating between the two species is possible, crosses between *H*. *medicinalis* and *H*. *verbana* results in a low fecundity with a high mortality of hybrid offspring [24]. We also note that while previous transcriptome assemblies for medicinal leeches are labeled *H*. *medicinalis*, the sequenced organisms are instead likely *H*. *verbana* due to commercial labeling practices [25]. Regardless, our transcriptome is the first to incorporate the entire central nervous system (CNS) with next-generation sequencing and annotation.

The CNS of *H*. *verbana* consists of 21 connected segmental ganglia comprising the ventral nerve cord, terminating at the cephalic end with a compound cephalic ganglion “brain”, and a caudal end with a compound terminal ganglion (“hind brain”) [26]. Each segmental ganglion is comprised of ~400 individual neurons, primarily as bilateral pairs surrounding the central neuropil [27]. The nervous system of a leech mediates a wide variety of stereotyped behaviors, from locomotor actions such as crawling [28] and swimming [29], to socialization [30] and prey localization [31].

Bringing Next-gen technologies to traditionally non-genetic invertebrate species has resulted in renewed focus on classical systems, such as the mollusk *Aplysia* (e.g., gill withdrawal reflex) [32] and crustaceans (e.g., motor rhythm generation in the stomatogastric nervous systems of *Homarus americanus* [33] and *Cancer borealis* [34]). Combining the experimental accessibility of these preparations with modern molecular techniques has facilitated the discovery of how such neural circuits function, and the medicinal leech is a prime model system to benefit from the same approach. In this study, we generated a *de novo* transcriptome assembly from the complete CNS of *H*. *verbana*. Our assembly generated 107,704 contigs from over 900 million raw reads. Of these 107,704 contigs, 39,047 (36%) were annotated using the validated RefSeq sequence database from NCBI (Bethesda, MD, USA). From this CNS transcriptome, we identified 126 unique ion channels, receptors, transporters, and enzyme contigs related to neural function and determined their relative abundance within the whole CNS. Not only is this a strong resource for investigating the molecular underpinnings of neural function in the medicinal leech, but it will also provide a reference for comparative analyses across taxa.

## Materials and methods

### Tissue collection and RNA preparation

Tissues used in this study were acquired from one adult *H*. *verbana* leech from Niagara Medical Leeches (Niagara Falls, NY, USA). Using only one animal potentially reduces the effect of single-nucleotide polymorphisms (SNPs) on the *de novo* transcriptome assembly [35]. Animals were maintained in ~10 gallon aquaria at 22–24°C using freshwater obtained from Lefevre Pond, Columbia, MO. Prior to dissection, the animal was anesthetized on ice for 10 min. For generation of the whole CNS transcriptome, the compound cephalic ganglion (including the non-segmental dorsal supraesophageal ganglion) through the compound terminal ganglion was removed from one animal by dissecting away the ventral sinus and anterior/posterior roots, leaving the connectives between ganglia intact. The entire ventral nerve cord was placed in 750 μL Trizol lysis buffer (Invitrogen, Carlsbad, CA, USA) and homogenized via a PowerGen 125 (Thermo Fisher Scientific, Waltham, MA, USA) set to high (5–6) until visible homogenization of nervous tissues was observed. Tissues were stored at -80° C until RNA extraction. Following the manufacturer’s protocol (Invitrogen), phenol-chloroform extraction was used to extract total RNA, with a subsequent DNase I treatment on Quick RNA Micro-prep Kit IC columns (Zymo Research, Irvine, CA, USA) to eliminate contamination from genomic DNA. A Nanodrop-1000 Spectrophotometer (Thermo Fisher Scientific) was used to determine purity and amount of total RNA.

### Library construction, sequencing, and *de novo* transcriptome assembly

Library construction and high-throughput sequencing services were performed by the University of Missouri DNA Core Facility (Columbia, MO, USA). Briefly, cDNA libraries were generated using the TruSeq Stranded mRNA Library Prep Kit (Illumina, San Diego, California, USA). Libraries were sequenced on the Illumina NextSeq 500 instrument in a 2 x 75 bp paired-end configuration. Fastq files generated from sequencing resulted in 919,249,178 total reads. Raw reads were trimmed with the Trimmomatic software package [36] to remove low quality bases, resulting in 897,014,584 clean reads. Trimmed fastq files were assembled into reference transcriptomes through two separate *de novo* assemblers for comparison: Trinity [37] (v2.4.0) and SeqMan NGen from the DNAstar software suite (SeqMan NGen®. Version 13.0. DNASTAR. Madison, WI.). Trinity *de novo* assembly yielded 107,704 contigs with a cutoff size of 200bp from 146,860,824 assembled bases, while SeqMan NGen generated only 64,565 contigs from 51,631,925 bases with the same cutoff.

### Transcriptome quality assessment

To determine the quality of the Trinity and SeqMan NGen *de novo* whole nervous system transcriptome assemblies, a Benchmarking Universal Single-Copy Orthologs (BUSCO) analysis was carried out on each transcriptome. The BUSCO analysis determines the number of complete, fragmented, or missing orthologs present in the transcriptome relative to the amount expected in the phylogenetic clade to which the organism is most closely related [38]. The *H*. *verbana* nerve cord transcriptome assemblies were compared against the Nematode and Arthropod databases. Complete orthologs are defined as being within 2 standard deviations in length from the ortholog in the database; fragmented orthologs are matches that fall outside the 95% expectation in length; missing orthologs failed to find a match in the transcriptome.

The sequence length distributions were also compared between assembled transcriptomes to determine which assembly produced longer, higher quality contigs more often. Given the significantly larger number of contigs generated by the Trinity build, and the inherent advantages of its open-source software, all further analyses were done using the Trinity assembly. Following contamination analysis carried out by NCBI, 161 contigs were excluded from the final transcriptome for similarity to known bacterial sequences.

### Sequence identification

Using known sequence information from NCBI, amino acid sequences for ion channel and receptor protein families were downloaded for *Drosophila melanogaster*, *Mus musculus*, and *Aplysia californica* and used as references for identifying homologous *H*. *verbana* sequences. The NCBI BLAST+ software suite (version 2.7.1) was used to generate a “BLASTable” database of contigs out of the Trinity assembled *H*. *verbana* CNS transcriptome. Reference amino acid sequences were used in a query with TBLASTN alignment of protein sequence against the translated nucleotide database of contigs. Putative hits underwent a second round of identity confirmation through a reverse BLASTX against the NCBI non-redundant (nr) database for all species as further validation. Identified contigs were named based on the BLAST hit with the highest score, and numerical values were assigned based on the order of identification for each given gene family. That is to say, we do not mean to imply specific membership positions within each gene subfamily, but rather simply provide a unique identifier to each sequence based on the order that it was identified in the transcriptome.

### Multiple sequence alignment and gene identification

Predicted coding sequences from *H*. *verbana* were identified from NCBI’s Open Reading Frame finder (ORFfinder) tool (https://www.ncbi.nlm.nih.gov/orffinder/) using the standard genetic code and default minimal ORF nucleotide length. Amino acid sequences were translated from the longest ORF to be used in multiple sequence alignment (MSA) to compare gene families across species. *H*. *verbana* amino acid sequences were compared against that of *Mus musculus*, *Drosophila melanogaster*, and *Aplysia californica*. MSA was carried out using NCBI’s Constraint Based Multiple Alignment (COBALT) tool (https://www.ncbi.nlm.nih.gov/tools/cobalt/) using default parameters. COBALT was chosen over other MSA tools because it incorporates conserved protein domain information into the alignments, beyond simply comparing amino acid sequence independently [39]. Phylogenetic trees were then generated using tree method set to cobalt tree and a max seq distance set to 0.85 with midpoint rooting.

### Gene ontology annotation

For assignment of gene ontology (GO) terms, the software package Blast2GO [40] (v5.0) was employed to functionally annotate the contigs of the Trinity assembled transcriptome. Using NCBI’s validated RefSeq database of Animalia protein sequences, a fast-BLASTX search (E-value threshold = 10^−5^) returned hit scores against aligned validated sequences. Descriptions were assigned to each contig based on the BLAST Top-hit out of the 20 significant hits for each contig (score threshold ≥ 55). GO term assignment produces multiple levels of GO terms, with the broadest ontological classifications being biological process, molecular function, and cellular component [41]. Assignment of GO terms included an E-value threshold = 10^−5^, GO weight = 5, and annotation cutoff = 55. GO term annotation, unique contig identifiers, sequence lengths, and other associated annotation information can be found in S1 File.

### RNA-seq TPM abundances

For quantitation of RNA-seq abundances for the transcripts investigated in this study, the software package Kallisto [42] (v0.43.1) was used to generate pseudo-alignments of the hundreds of millions of paired-end reads from the fastq files generated in the sequencing of the CNS of *H*. *verbana* against the coding sequences of identified genes of interest from the Trinity assembled transcriptome. Abundances were normalized using the transcripts per kilobase million (TPM) method, which accounts for contig length in kilobases and the number of millions of reads aligned.

## Results

### CNS sequencing and *de novo* transcriptome assembly

**Fig 1:**
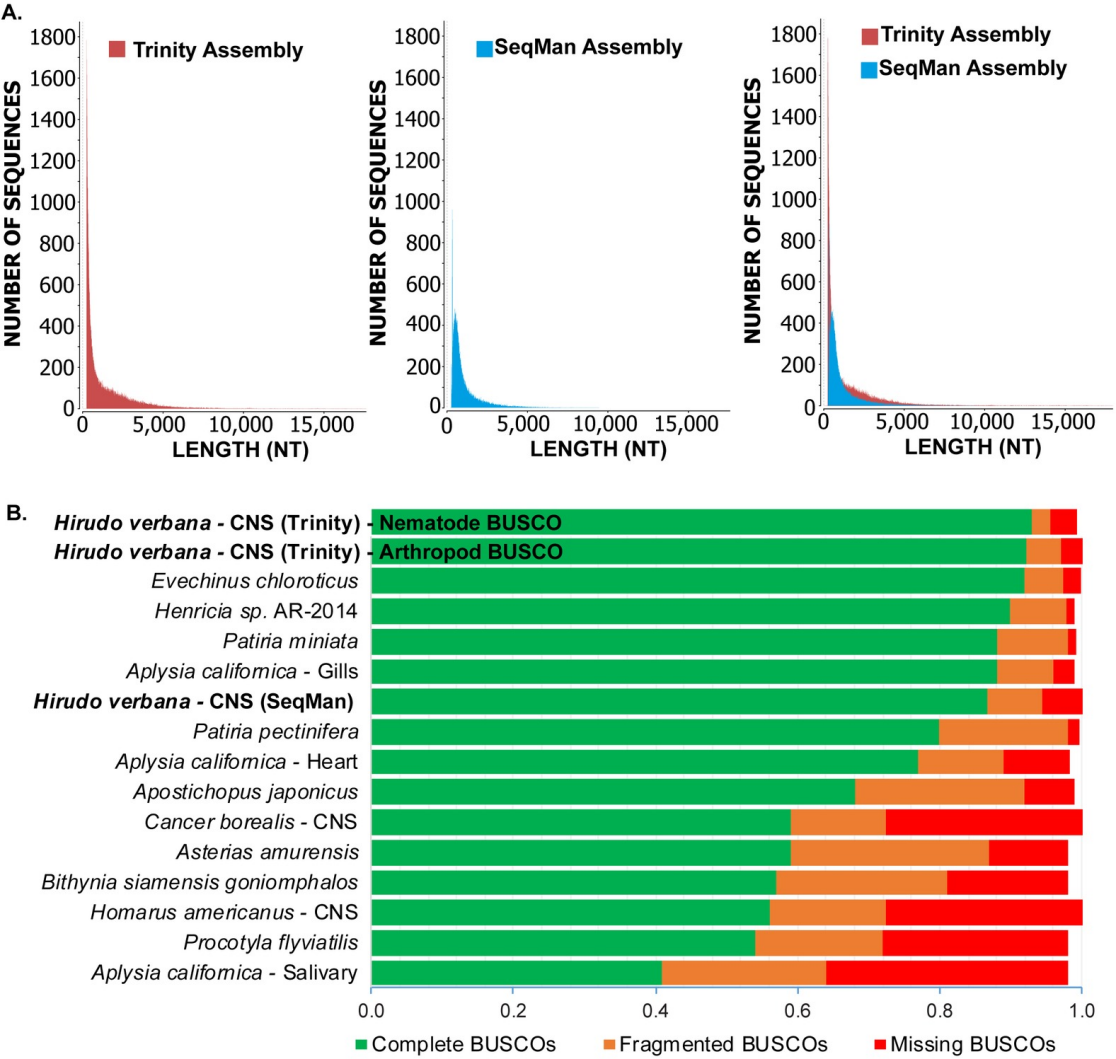
Transcriptome quality-assessment comparison between Trinity and SeqMan *de novo* assembled transcriptomes. (A). Size distribution of assembled contigs for each *de novo* assembly of the *H*. *verbana* CNS transcriptome. Overlaying the size distributions allows for direct comparison of contig sizes produced by each software. (B). Benchmarking Universal Single-Copy Orthologs (BUSCO) quality categories are horizontally stacked in bar plots as a quality comparison among assembled transcriptomes both across species and tissues. Assemblies produced in this publication (Trinity and SeqMan) are indicated in bold. Previously published CNS system transcriptomes from *C*. *borealis* and *H*. *americanus* were added for comparison with the same tissue type, which was lacking from the Nematoda BUSCO database.

From the sequencing of the entire CNS of *H*. *verbana*, 919,249,178 raw reads were generated from 75 bp paired-end Illumina sequencing. Filtering of reads resulted in 897,014,584 clean reads with over 97% of reads with a quality score greater or equal to 30. These high-quality reads were used in the *de novo* assembly of transcriptomes using two separate transcriptome assembly software packages. The two assemblers used in this study were the Trinity (v2.4.0) software package (comprised of Inchworm, Chrysalis, and Butterfly [43]) and SeqMan NGen from the DNAstar’s Lasergene software package [44]. The Trinity assembly yielded 107,704 contiguous sequences (contigs), comprising 146,860,824 total bases, with an N_50_ of 2544bp and mean contig length of 1363bp. The SeqMan assembly resulted in 64,565 contigs from 51,631,692 total bases with an N_50_ of 1286 and mean length of 800bp (Table 1). When the sequence length distributions are compared, the Trinity assembly not only has more total sequences, but particularly has more sequences of a greater length than that of the SeqMan assembly (Fig 1A).

**Table 1:** CNS transcriptome sequencing and assembly statistics.

	*H*. *verbana*	
Raw reads	919,249,178	
Clean reads	897,014,584	
% Q Scores ≥ 30	97.58	
% GC	41	
Assemblers	Trinity	SeqMan NGen
Number of Contigs	107,704	64,565
N_50_ (bp)	2544	1286
Mean contig length (bp)	1363	800
Longest contig (bp)	35,088	8773
Total assembled bases	146,860,824	51,631,925

To compare the quality of the Trinity transcriptome against the SeqMan transcriptome, the Benchmarking Universal Single-Copy Ortholog (BUSCO) software was used to assess the transcriptome completeness based on the presence of expected lineage-specific orthologs [38]. At the time of this publication, the Nematoda BUSCO reference contained 978 identified universal single-copy orthologs for assessing genomic and transcriptomic assembly quality. The BUSCO analysis reports how many of these orthologs were found to be complete, fragmented, or missing in the queried assembly. The Trinity assembly of the *H*. *verbana* CNS transcriptome resulted in 93.6% complete, 2.9% fragmented, and 3.5% missing BUSCOs, while the SeqMan assembly resulted in 86.7% complete, 7.8% fragmented, and 5.5% missing BUSCOs (Fig 1B). Both transcriptome assemblies performed well compared to BUSCO assessments from other species, including both tissue-specific and whole organism transcriptomes. Particularly, the Trinity assembled *H*. *verbana* transcriptome had the greatest percentage of complete BUSCOs out of the references in the database. We also included two BUSCO assessments for previously published arthropod CNS transcriptomes from *Cancer borealis* and *Homarus americanus* [34] to compare BUSCO scores in a tissue specific manner, since the Nematoda references were missing comparable nervous system tissues.

Based on the transcriptome assembly statistics, sequence length distribution comparison, and BUSCO quality metrics, we decided to proceed with the Trinity transcriptome in annotation and analysis and did not move forward with the SeqMan transcriptome, which all metrics indicated was lower quality. The *H*. *verbana* Trinity assembled Transcriptome Shotgun Assembly (TSA) project has been deposited at DDBJ/ENA/GenBank (BioProject No. PRJNA435743; BioSample No. SAMN08595902) under the accession GGIQ00000000. The version described in this paper is the first version, GGIQ01000000. The Sequence Read Archive (SRA) can be found at SRR6782848.

### Sequence identification and gene ontology annotation

**Fig 2:**
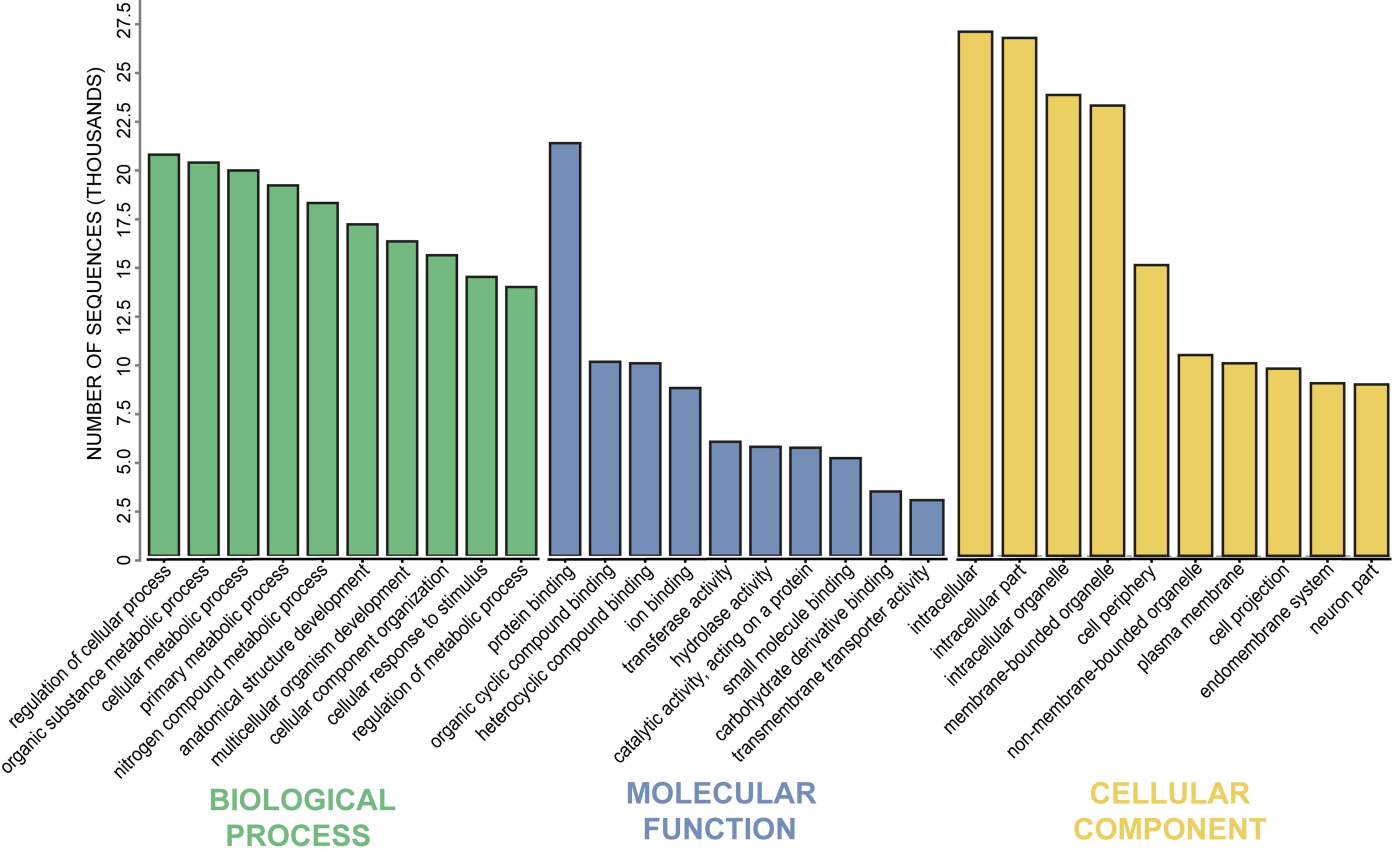
Gene ontology distribution of the annotated *H*. *verbana* CNS transcriptome. The absolute values of the top 10 most abundant sequence annotations for each classification are represented at a GO level of 3. Major GO categorizations are classified into biological process, molecular function, and cellular component.

Using the Blast2GO software suite [40], a fast-blastx alignment was carried out against NCBI’s validated RefSeq Animalia database to identify and annotate contigs from the *H*. *verbana* CNS transcriptome. Out of the 107,704 contigs in the transcriptome, 39,047 contigs were annotated with a BLAST hit and sequence descriptor. Following BLASTing of the transcriptome, gene ontology mapping was carried out to assign GO terms to the contigs that returned a BLAST hit. The top 10 GO terms for each of the major GO classifications (biological process, molecular function, and cellular component) at a GO level of 3 are represented in Fig 2. This GO level was chosen as it balances returning broad GO terms while still being relevant to the specific tissue type from which the transcriptome was assembled. Examples of this can be seen in the GO terms reported in the cellular component classification, such as “cell projection” and “neuron part”, as well as in molecular function in “transmembrane transporter activity”. This annotated reference transcriptome is available for studies examining GO terms of interest from more specific levels than what is presented here.

### Species hit distribution

**Fig 3:**
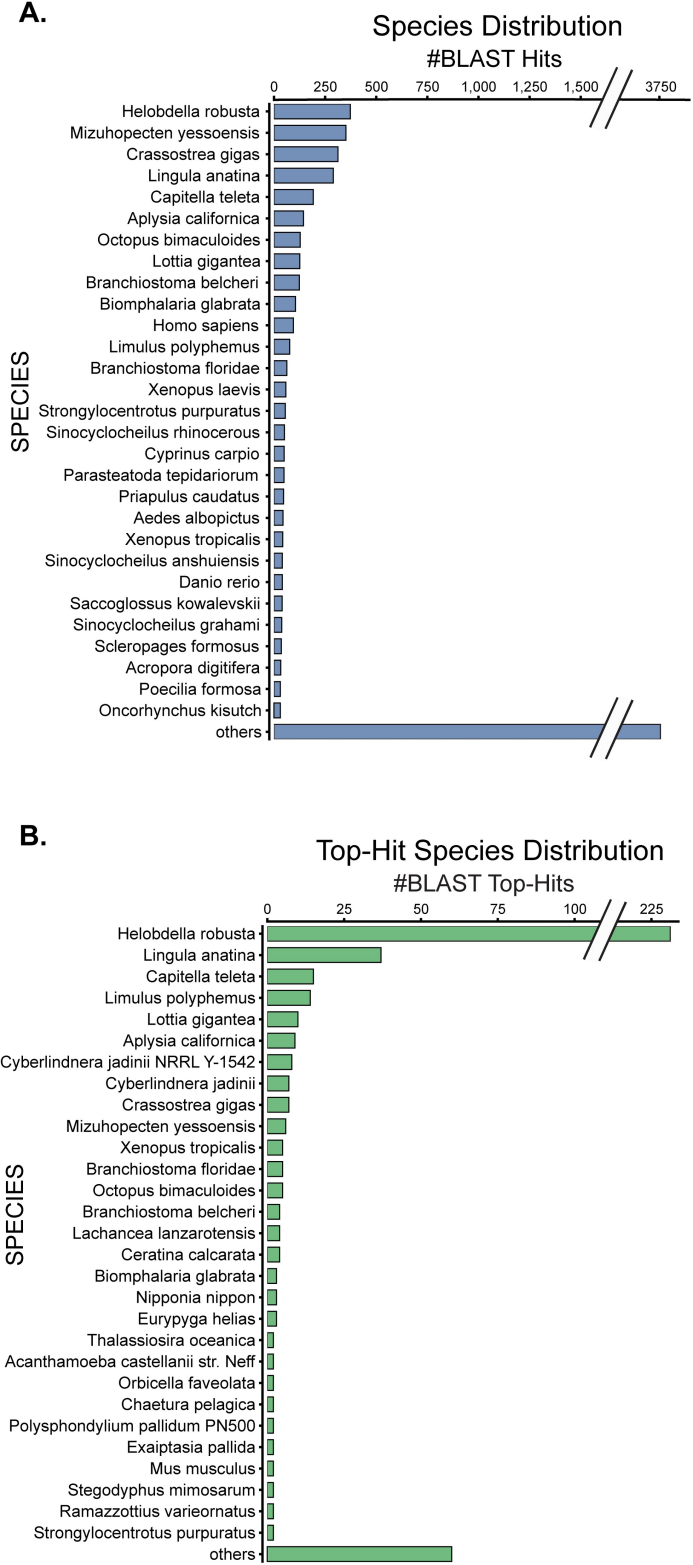
Blast hit species distribution of *H*. *verbana* nervous system transcriptome. (A). Frequency of species assigned to each contig from total BLAST hits (up to 20 hits per contig) against NCBI’s validated RefSeq database of Animalia protein sequences with an e-value threshold = 10^−5^. (B). Frequency of species assigned to each contig from the highest scoring BLAST hit for each contig.

In the annotation of the *H*. *verbana* CNS transcriptome, we captured the distribution of BLAST hits that corresponded to specific species (Fig 3). We separated the distributions into total BLAST hits, which includes up to 20 BLAST results for each contig, and top BLAST hit, which is the single hit with the highest score for that contig. The top species for both distributions was by far the California leech *Helobdella robusta*, which is unsurprising as both organisms belong to the sub-class Hirudinea within the clitellate annelids. Another annelid worm, *Capitella teleta*, was in the top 5 species for each distribution. Other notable species included the brachiopod *Lingula anatine*, as well as many members of the phylum Mollusca: the giant owl limpet *Lottia gigantea*, Pacific oyster *Crassostrea gigas*, and California sea slug *Aplysia californica*. With *Aplysia* being ranked 6^th^ in each distribution, as well as having a well-annotated nervous system transcriptome [45], we compared the previously undiscovered ion channel and receptor contigs mined from our transcriptome to those of *Aplysia*, as well as *M*. *musculus* and *D*. *melanogaster*.

### Innexins

Since the innexin gap-junction proteins have been well-characterized in *H*. *verbana* from genomic sequencing [12], we used innexins as an additional benchmarking tool for assessing transcriptome quality by comparison of innexin contigs from our transcriptome against published innexin sequences, as shown in Table 2. At the nucleotide level, our contigs matched the published innexin sequences with high fidelity (average 99.8% nucleotide identity). Independently arriving at near 100% nucleotide identity from over 20 genomic and transcriptomic comparisons provided us strong confidence in the quality of our transcriptome assembly. We further performed a basic comparison of innexin presence in the leech CNS by comparing the RT-PCR results from Kandarian et al. 2012 to RNA-seq counts from our transcriptome (Table 2). The results coincide moderately well, with innexins having higher RNA-seq counts producing positive bands in the previous RT-PCR results, and those with lower RNA-seq counts not detected by RT-PCR [12].

**Table 2:** Comparison of previously described innexin sequence identity and expression against CNS transcriptome.

Gene name	EST Genome Accession #	PCR in CNS [12]	Seq counts	Nucl % Identity	CNS Transcriptome Contig
INX1	JQ231005.1	+	29594	99.8	TRINITY_DN32921_c2_g1_i1
INX2	JQ231006.1	+	112620	99.7	TRINITY_DN27867_c1_g1_i3
INX3	JQ231007.1	+	40545	99.9	TRINITY_DN36662_c5_g1_i2
INX4	JQ231008.1	+	44693	99.8	TRINITY_DN32766_c0_g1_i2
INX5	JQ231009.1	-	11011	100	TRINITY_DN34224_c2_g3_i6
INX6	JQ231010.1	+	3469	100	TRINITY_DN30941_c0_g1_i1
INX7	JQ231011.1	-	2560	99.6	TRINITY_DN34351_c2_g2_i5
INX8	JQ231012.1	-	799	99.8	TRINITY_DN26342_c0_g1_i1
INX9A	JQ231013.1	+	64313	99.8	TRINITY_DN30681_c0_g1_i3
INX9B	JQ231014.1	+	320800	99.8	TRINITY_DN30681_c0_g1_i1
INX10	JQ231015.1	-	333	100	TRINITY_DN25776_c0_g2_i1
INX11A	JQ231016.1	-	32920	99.9	TRINITY_DN35052_c5_g2_i13
INX11B	JQ231017.1	-	3130	99.7	TRINITY_DN35052_c5_g2_i7
INX12	JQ231018.1	+	119237	99.8	TRINITY_DN35652_c1_g4_i3
INX13	JQ231019.1	-	25630	98.3	TRINITY_DN33966_c3_g4_i9
INX14	JQ231020.1	+	58726	99.6	TRINITY_DN34885_c3_g1_i3
INX15	JQ231021.1	+	16988	99.8	TRINITY_DN32315_c5_g5_i3
INX16	JQ231022.1	+	32534	100	TRINITY_DN29870_c0_g1_i2
INX17	JQ231023.1	+	66718	100	TRINITY_DN20318_c0_g1_i2
INX18	JQ231024.1	+	11855	100	TRINITY_DN35830_c3_g1_i5
INX19	JQ231025.1	+	1525	100	TRINITY_DN33800_c3_g1_i1

### Ion channels

In mining the *H*. *verbana* CNS transcriptome for sequences related to nervous system function, our first targets were ion channels. Our first-pass approach to mining the transcriptome yielded contigs that putatively belong to subfamilies of ion channels, but we cannot say with certainty that each contig represents a single unique gene. Rather, we report here the number of discrete, nonoverlapping contigs identified from our de novo transcriptome that could not be further combined based on assembly sequence alone. The contigs reported here lay the foundation for further more detailed characterization. In total, we identified 40 potassium (K^+^) channels contigs, 5 transient receptor potential (TRP) channel contigs, 4 cyclic nucleotide-gated channel contigs, 10 calcium (Ca^2+^) channel contigs, and 4 sodium (Na^+^) channel contigs. Comparing the ion channels pulled from the transcriptome against that of *A*. *californica*, *M*. *musculus*, and *D*. *melanogaster* provided additional confidence in our identification. Ion channel subtypes with descriptions of the putative currents known to be generated by their orthologs, as well as their NCBI accession numbers, are reported in Tables 3 and 4.

**Table 3:** K^+^, TRP, and CNG channel putative currents and accession numbers from CNS transcriptome.

Channel Family	Gene Name	Current/Channel Type	*H*. *verbana* accession
**Voltage-dependent** **K**^**+**^ **Channels**	*Shaker1*	Voltage-gated A-type potassium (I_A_ or K_v_1)	MG973375
	*Shaker2*	Voltage-gated A-type potassium (I_A_ or K_v_1)	MG973376
	*Shaker3*	Voltage-gated A-type potassium (I_A_ or K_v_1)	MG973377
	*Shaker4*	Voltage-gated A-type potassium (I_A_ or K_v_1)	MG973378
	*Shab1*	Voltage-gated delayed rectifier (I_Kd_ or K_v_2)	MG973379
	*Shab2*	Voltage-gated delayed rectifier (I_Kd_ or K_v_2)	MG973383
	*Shab3*	Voltage-gated delayed rectifier (I_Kd_ or K_v_2)	MG973384
	*Shab4*	Voltage-gated delayed rectifier (I_Kd_ or K_v_2)	MG973385
	*Shab5*	Voltage-gated delayed rectifier (I_Kd_ or K_v_2)	MG973387
	*Shaw1*	Voltage-gated delayed rectifier (I_Kd_ or K_v_3)	MG973386
	*Shaw2*	Voltage-gated delayed rectifier (I_Kd_ or K_v_3)	MG973388
	*Shaw3*	Voltage-gated delayed rectifier (I_Kd_ or K_v_3)	MG973389
	*Shaw4*	Voltage-gated delayed rectifier (I_Kd_ or K_v_3)	MG973390
	*Shal1*	Voltage-gated A-type potassium (I_A_ or K_v_4)	MG973380
	*Shal2*	Voltage-gated A-type potassium (I_A_ or K_v_4)	MG973381
	*Shal3*	Voltage-gated A-type potassium (I_A_ or K_v_4)	MG973382
	*KCNQ1*	Voltage-gated slow delayed rectifier (M-type or K_v_7)	MG973372
	*KCNQ2*	Voltage-gated slow delayed rectifier (M-type or K_v_7)	MG973373
	*KCNQ3*	Voltage-gated slow delayed rectifier (M-type or K_v_7)	MG973374
	*KCNH1*	Ether-a-go-go type potassium	MG973391
	*KCNH2*	Ether-a-go-go type potassium	MG973392
	*KCNH3*	Ether-a-go-go type potassium	MG973393
	*KCNH4*	Ether-a-go-go type potassium	MG973394
	*KCNH5*	Ether-a-go-go type potassium	MG973395
	*KCNH6*	Ether-a-go-go type potassium	MG973396
	*KCNH7*	Ether-a-go-go type potassium	MG973397
	*KCNH8*	Ether-a-go-go type potassium	MG973398
**Other K**^**+**^ **channels**	*BKKCa1*	Large conductance (BK) voltage/Ca^2+^-activated potassium	MG973418
	*BKKCa2*	Large conductance (BK) voltage/Ca^2+^-activated potassium	MG973419
	*BKKCa3*	Large conductance (BK) voltage/Ca^2+^-activated potassium	MG973420
	*SKKCa1*	Small conductance (SK) Ca^2+^-activated potassium	MG973364
	*SKKCa2*	Small conductance (SK) Ca^2+^-activated potassium	MG973365
	*SKKCa3*	Small conductance (SK) Ca^2+^-activated potassium	MG973366
	*KCNT*	Sodium-activated potassium	MG973421
	*IRK1*	Inward-rectifier potassium (IRK)	MG973360
	*IRK2*	Inward-rectifier potassium (IRK)	MG973361
	*IRK3*	Inward-rectifier potassium (IRK)	MG973362
	*IRK4*	Inward-rectifier potassium (IRK)	MG973363
	*KCNK*	Two-pore domain leak potassium (K_2p_)	MG973422
**Transient Receptor Potential (TRP) Channels**	*TRP1*	Transient receptor potential cation	MG973367
	*TRP2*	Transient receptor potential cation	MG973368
	*TRP3*	Transient receptor potential cation	MG973369
	*TRP4*	Transient receptor potential cation	MG973370
	*TRP5*	Transient receptor potential cation	MG973371
**Cyclic** **Nucleotide Gated** **Channels**	*CNG1*	Cyclic nucleotide-gated	MG973356
	*CNG2*	Cyclic nucleotide-gated	MG973357
	*CNG3*	Cyclic nucleotide-gated	MG973358
	*CNG4*	Cyclic nucleotide-gated	MG973359

**Table 4:** Ca^2+^ and Na^+^ channel putative currents and accession numbers from CNS transcriptome.

Channel Family	Gene Name	Current/Channel Type	*H*. *verbana* accession
**Ca**^**2+**^ **Channels**	*CaV-I*	Voltage-dependent calcium	MG973400
	*CaV-II*	Voltage-dependent calcium	MG973401
	*CaV-III*	Voltage-dependent calcium	MG973402
	*CaV-IV*	Voltage-dependent calcium	MG973403
	*CaV-V*	Voltage-dependent calcium	MG973404
	*CaV-VI*	Voltage-dependent calcium	MG973405
	*CaV-VII*	Voltage-dependent calcium	MG973406
	*CaV-VIII*	Voltage-dependent calcium	MG973407
	*CaV-IX*	Voltage-dependent calcium	MG973408
	*CaV-X*	Voltage-dependent calcium	MG973409
**Na**^**+**^ **Channels**	*NaV-I*	Voltage-gated sodium	MG973410
	*NaV-II*	Voltage-gated sodium	MG973411
	*NaV-III*	Voltage-gated sodium	MG973412
	*NALCN*	Non-selective sodium leak	MG973399

**Fig 4:**
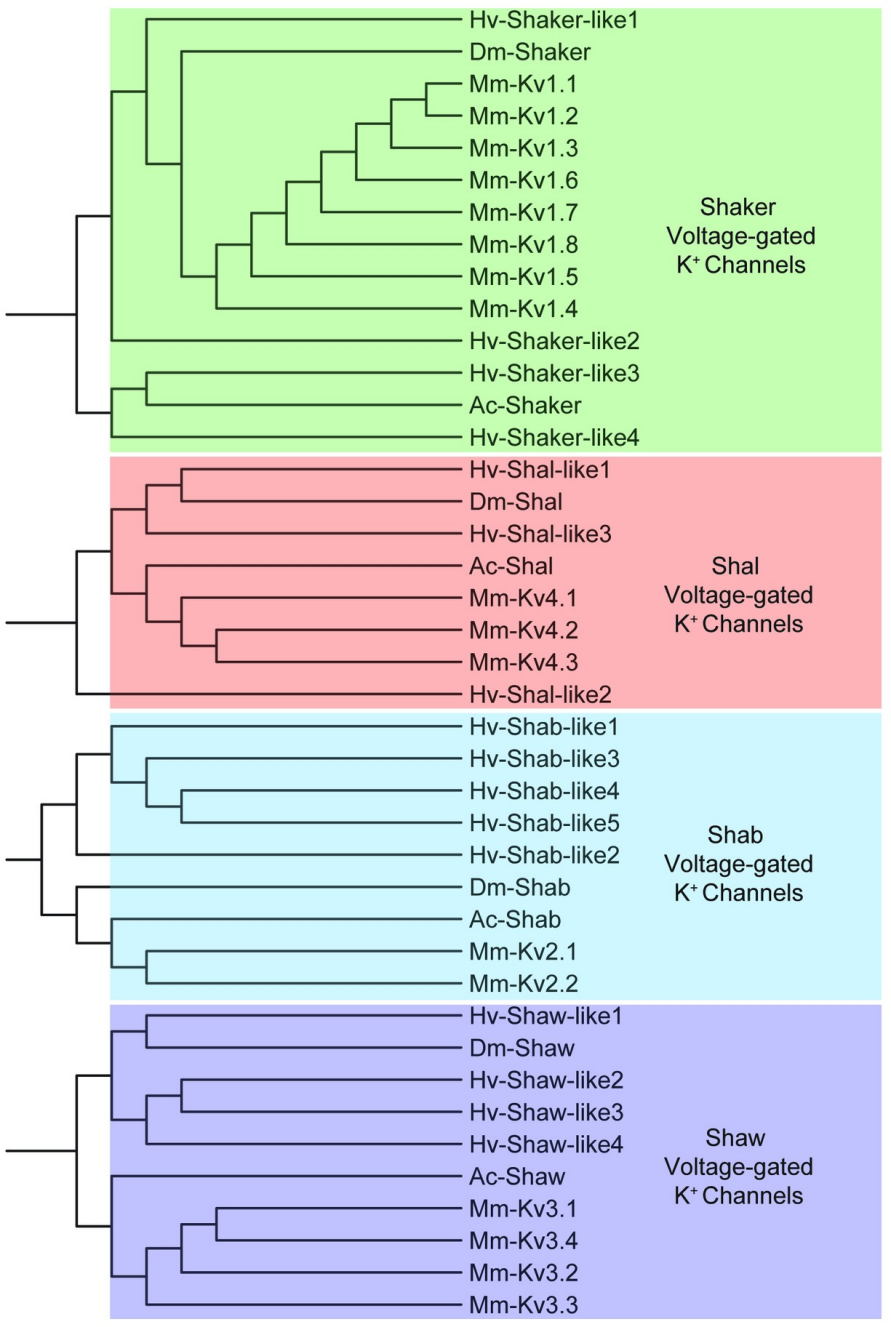
Voltage-gated K^+^ channel subfamilies identified in *H*. *verbana* CNS transcriptome. NCBI’s COBALT tool generated both amino acid sequence alignment and, subsequently, tree diagrams. These tree-based analyses are not meant to represent true phylogenetic relationships, but rather add another layer of confidence to the identification of putative gene-family subtypes; thus, bootstrap values were not analyzed. Transcript prefix identifications are as follows: “Hv” = *H*. *verbana*, “Mm” = *Mus musculus*, “Ac” = *Aplysia californica*, and “Dm” = *Drosophila melanogaster*. *H*. *verbana* voltage-gated K^+^ channel subtypes, putative membrane currents, and accession numbers can be found in Table 3.

**Fig 5:**
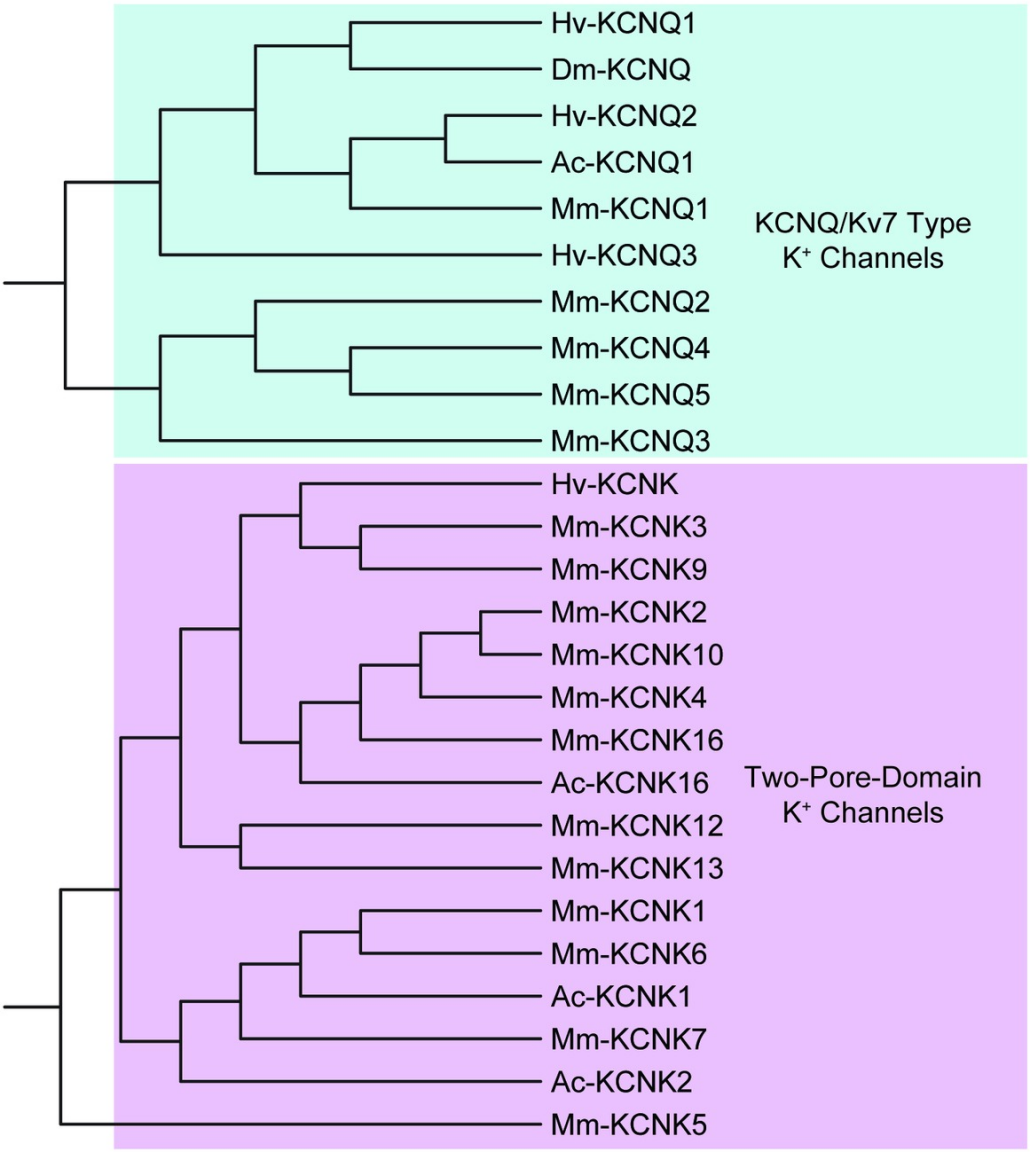
KCNQ and KCNK channel families identified in *H*. *verbana* CNS transcriptome. Trees were produced in the same manner as in Fig 4. *H*. *verbana* KCNQ and KCNK channel subtypes, putative membrane currents, and accession numbers can be found in Table 3.

For the voltage-gated K^+^ channel family, we identified 4 *shaker*-like, 5 *shab-*like, 4 *shaw-*like, and 3 *shal-*like K^+^ channel contigs, named according to their original identification in *Drosophila* [46]. The mammalian equivalents are *K*_*v*_*1*, *K*_*v*_*2*, *K*_*v*_*3*, and *K*_*v*_*4*, respectively. Clustering of these voltage-gated K^+^ channel conitgs resulted in discrete nodes separating the four subfamilies (Fig 4). In these clustering analyses, we do not mean to imply true phylogenetic relationships, but rather use this as a tool to provide additional confidence to our sequence identification (see Methods). Other voltage-gated K^+^ channel contigs identified from the *H*. *verbana* CNS transcriptome include three members of the slow delayed rectifier *KCNQ* (*K*_*v*_*7*) family and 8 members of the “*ether-a-go-go*” like *KCNH* family (Table 3). As stated in the methods, the numbering of each gene name corresponds to the order in which it was identified from our transcriptome, and not to unique membership positions within a subfamily. Also identified was one two-pore domain leak K^+^ (*K*_*2p*_) channel similar to the *KCNK* family of channels. As expected, clustering of *H*. *verbana KCNK* and *KCNQ* with that of *A*. *californica*, *D*. *melanogaster*, and *M*. *musculus* resulted in two major nodes for each subfamily (Fig 5).

**Fig 6:**
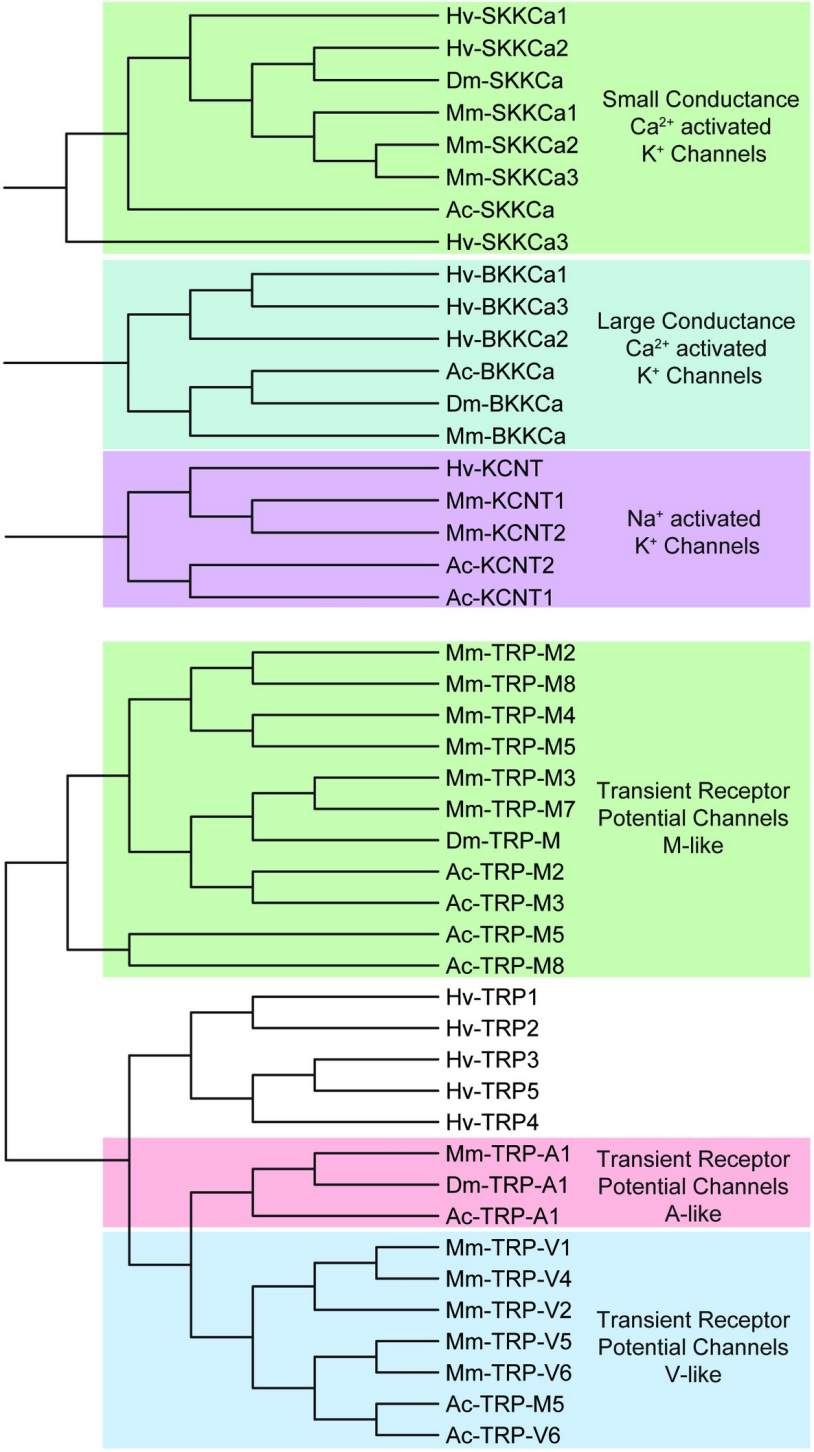
TRP, Ca^2+^-activated K^+^, and Na^+^-activated K^+^ channels identified in *H verbana* CNS transcriptome. Trees were produced in the same manner as in Fig 4. *H*. *verbana* TRP channels were more similar to A- or V-like than M-like, but could not be further described accurately, being equal nodes away from the A- and V-like clusters. *H*. *verbana* TRP, Ca^2+^-activated K^+^, and Na^+^-activated K^+^ channel subtypes, putative membrane currents, and accession numbers can be found in Table 3.

Other identified *H*. *verbana* K^+^ channel contigs included three large (BK) and three small (SK) conductance Ca^2+^-activated K^+^ channels labeled *BKKCa* and *SKKCa*, respectively. One Na^+^-activated K^+^ channel *KCNT* was also identified. The clustering of *BKKCa*, *SKKCa*, and *KCNT* from the four species resulted in three unique nodes in the clustering of these K^+^ channels (Fig 6A). The last of the other K^+^ channels identified was the inward rectifier K^+^ channel *IR*K, of which four sequences were found resembling this family (Table 3).

**Fig 7:**
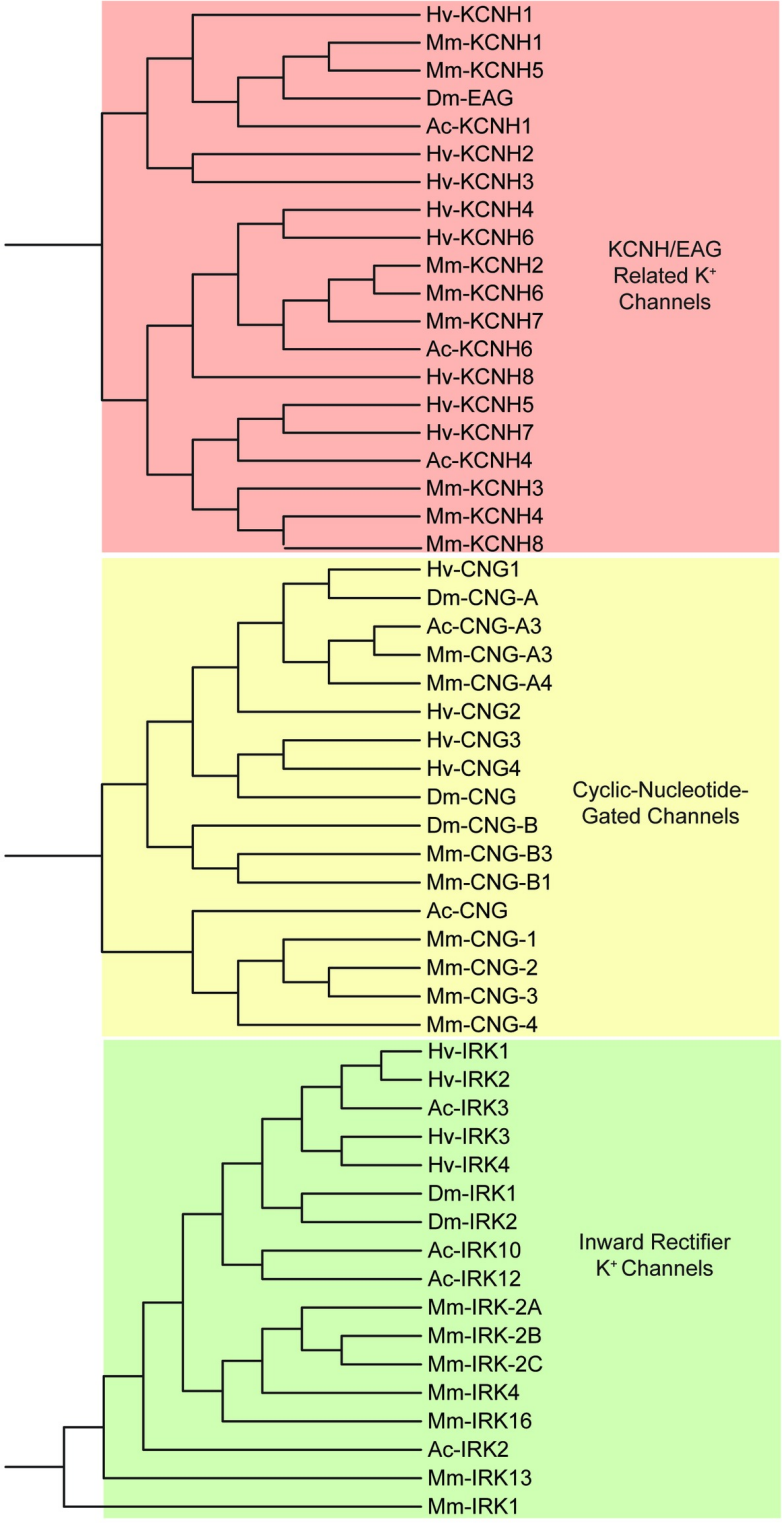
KCNH/EAG, CNG, and IRK channel families identified in *H*. *verbana* CNS transcriptome. Trees were produced in the same manner as in Fig 4. *H*. *verbana* KCNH/EAG, CNG, and IRK channel subtypes, putative membrane currents, and accession numbers can be found in Table 3.

For ion channels non-selectively permeable to cations, 4 cyclic nucleotide-gated (*CNG*) channel contigs and 5 transient receptor potential (*TRP*) channel contigs were identified in the CNS transcriptome of *H*. *verbana*. The *TRP* channels from *H*. *verbana* were compared against the *TRP-M* (Melastatin), *TRP-A* (Ankyrin), and *TRP-V* (Vanilloid) subtypes of *D*. *melanogaster*, *A*. *californica*, and *M*. *musculus* (Fig 6B). The clustering results indicated that the *H*. *verbana TRP* channels were not similar enough to assign a specific subtype, as they were equally distant to *TRP-A* and *TRP-V*. Clustering of the *CNG* channels along with *IRK* and *KCNH* separated out into three major nodes for each gene family (Fig 7).

**Fig 8:**
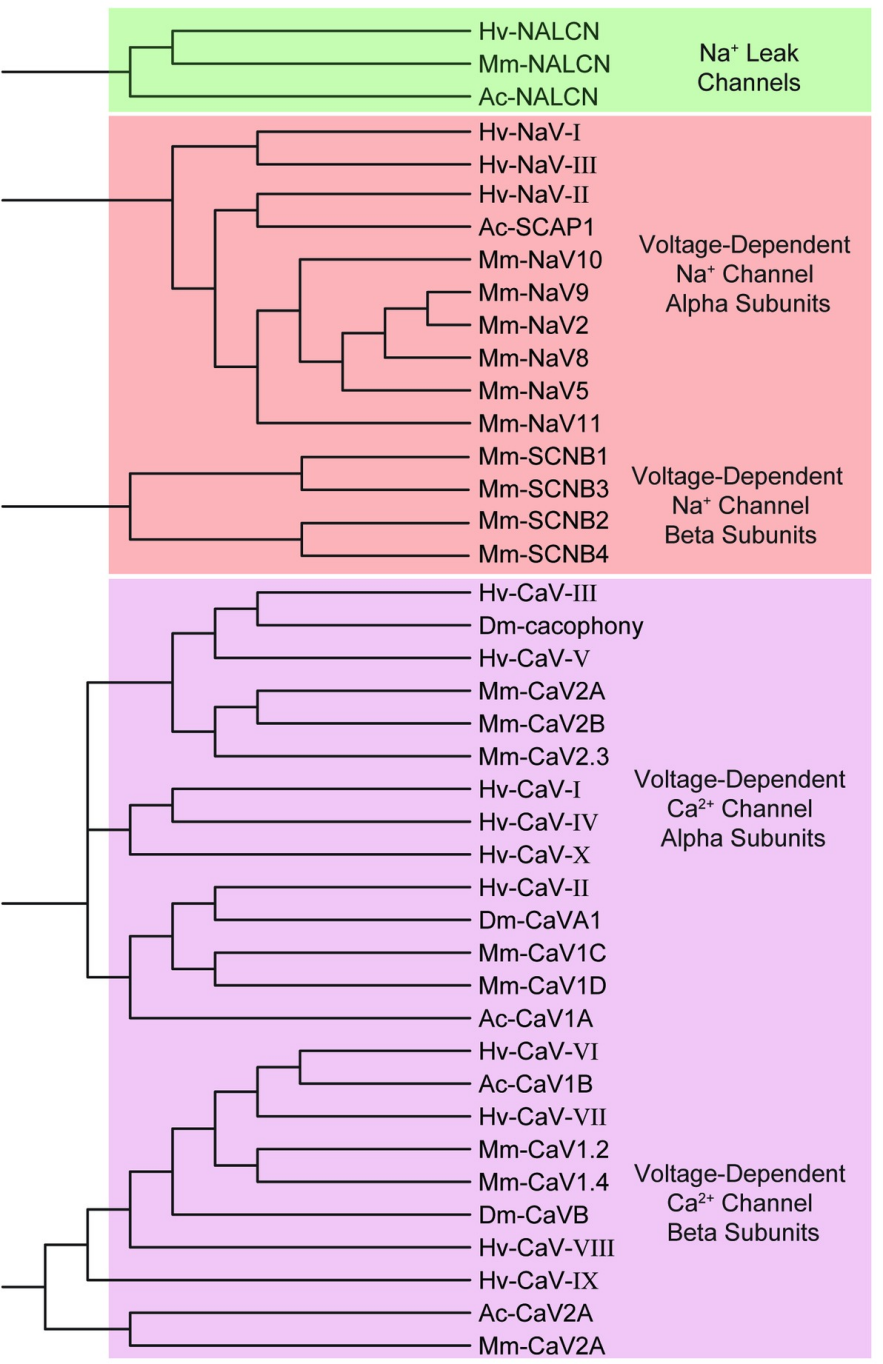
Families of sodium and calcium ion channel subtypes identified in *H*. *verbana* CNS transcriptome. Trees were produced in the same manner as in Fig 4. The putative *H*. *verbana* sodium and calcium ion channel subtypes are listed in Table 4 with accession numbers and putative associated membrane currents.

The voltage-gated Na^+^ and Ca^2+^ channel contigs identified from the *H*. *verbana* CNS transcriptome totaled 13 sequences: three voltage-gated Na^+^ and 10 voltage-gated Ca^2+^ channels (Table 4). Voltage-gated Na^+^ and Ca^2+^ channels were enumerated using roman numerals rather than numbers to avoid implying–for instance–that calcium channel one belonged to the *CaV1* family of voltage-gated calcium channels. One Na^+^ leak channel contig orthologous to *NALCN* was identified. This was expected as it has been shown that *NALCN* appears with only one copy in the vast majority of species studied[47]. Clustering of the voltage-gated Na^+^ and Ca^2+^ channels, as well as the Na^+^ leak channel, yielded an expected separation where the Na^+^ leak channels were more similar to the voltage-gated Na^+^ channels than voltage-gated Ca^2+^ channels (Fig 8).

### Comparison of ion channel content across leech species

Following identification of ion channels in the *H*. *verbana* CNS transcriptome, we carried out a similar methodology for predicted coding sequences from the genome of the Californian leech (*Helobdella robusta*) to determine if the number of ion channel contigs we identified is similar across species (Table 5).

**Table 5:** Number of identified sequences from *H*. *verbana* CNS transcriptome and *H*. *robusta* predicted genome CDS using the same gene identification approaches.

Channel Family	*H*. *verbana* Transcriptome	*H*. *robusta* Predicted Genome				*H*. *robusta* CDS IDs			
**Voltage-dependent** **K**^**+**^ **Channels**	27	34	HelroT121337 HelroT91264 HelroT65928 HelroT72163 HelroT84384	HelroT141211 HelroT130121 HelroT79879 HelroT162251 HelroT75893	HelroT122508 HelroT168092 HelroT122624 HelroT66265 HelroT62501	HelroT67024 HelroT108240 HelroT87691 HelroT119837 HelroT157793	HelroT119855 HelroT87727 HelroT66677 HelroT162328 HelroT86941	HelroT94060 HelroT64770 HelroT63495 HelroT81288 HelroT69639	HelroT122109 HelroT75592 HelroT78253 HelroT64112
**Other K**^**+**^ **channels**	12	17	HelroT168799 HelroT119046 HelroT112655	HelroT64618 HelroT63354 HelroT158811	HelroT119254 HelroT108545 HelroT176931	HelroT111253 HelroT82781	HelroT106279 HelroT73033	HelroT122071 HelroT122255	HelroT79786 HelroT123209
**TRP Channels**	5	7	HelroT85683	HelroT63393	HelroT169548	HelroT130200	HelroT191443	HelroT66128	HelroT191443
**CNG** **Channels**	4	6	HelroT90895	HelroT175626	HelroT162466	HelroT71626	HelroT71626	HelroT86050	HelroT82034
**Ca**^**2+**^ **Channels**	10	11	HelroT73530 HelroT161162	HelroT128993 HelroT160604	HelroT119050 HelroT170765	HelroT68810 HelroT148954	HelroT128998	HelroT161161	HelroT160605
**Na**^**+**^ **Channels**	4	8	HelroT123810 HelroT155980	HelroT119038	HelroT89291	HelroT109965	HelroT71177	HelroT64539	HelroT87061

Overall, relatively similar sequence numbers were found between the two species when the same methods were employed; however, more putative sequences belonging to ion channel families were determined to be present in the predicted CDS from the genome of *H*. *robusta* as compared to the CNS transcriptome of *H*. *verbana*. This difference is potentially due to the transcriptome of *H*. *verbana* representing only CNS tissue, whereas the predicted CDS of *H*. *robusta* reflects the full genome. The genome sequence may also reveal ion channel pseudogenes that are not actually expressed. The corresponding *H*. *robusta* predicted CDS ID numbers can be found in Table 5.

### Biogenic amine receptors

**Fig 9:**
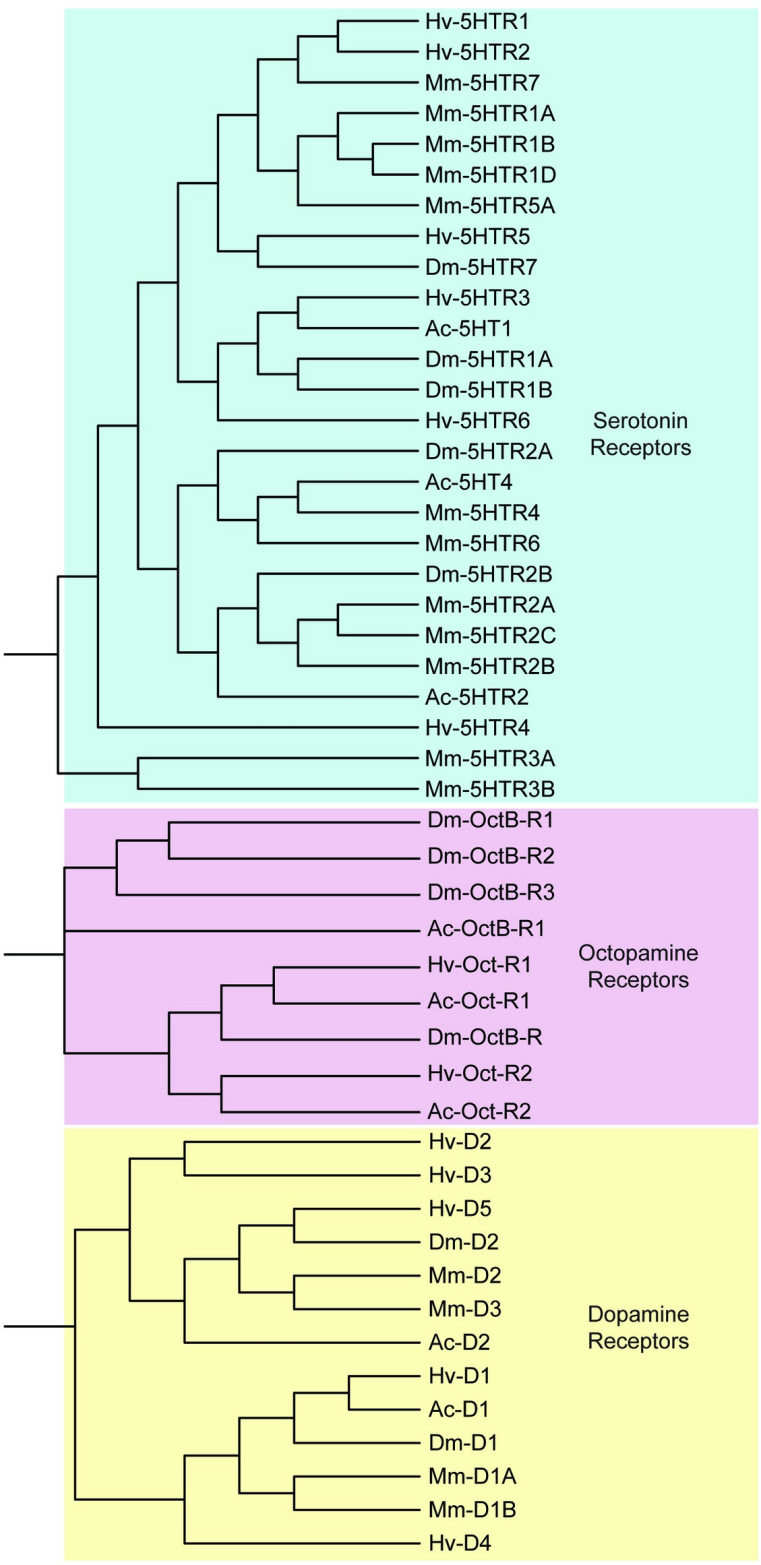
Biogenic amine receptor families identified in the *H*. *verbana* CNS transcriptome. Trees were produced in the same manner as in Fig 4. *H*. *verbana* biogenic amine receptor subtypes and accession numbers can be found in Table 5.

The biogenic amine receptor contigs identified from the *H*. *verbana* CNS transcriptome included 5 dopamine (*D1-5*), 6 serotonin (5HTR1-6*)*, and two octopamine (*Oct-R1-2*) receptor subtypes (Table 6). Clustering of biogenic amine receptors resulted in three clear nodes that corresponded to each type of receptor (Fig 9). No norepinephrine receptors were detected in the leech transcriptome, consistent with previous physiological reports [48].

**Table 6:** Biogenic amine and GABA receptor accession numbers from CNS transcriptome.

Receptor Family	Gene Name	*H*. *verbana accession*
**Octopamine Receptors**	*Oct1*	MG973328
	*Oct2*	MG973329
**Dopamine Receptors**	*D1*	MG973317
	*D2*	MG973318
	*D3*	MG973319
	*D4*	MG973320
	*D5*	MG973321
**Serotonin Receptors**	*5HTR1*	MG973322
	*5HTR2*	MG973323
	*5HTR3*	MG973324
	*5HTR4*	MG973325
	*5HTR5*	MG973326
	*5HTR6*	MG973327
**GABA Receptors**	*GABAr1*	MG973330
	*GABAr2*	MG973331
	*GABAr3*	MG973332
	*GABAr4*	MG973333
	*GABAr5*	MG973334
	*GABAr6*	MG973335
	*GABAr7*	MG973336
	*GABAr8*	MG973337
	*GABAr9*	MG973338
	*GABAr10*	MG973339
	*GABAr11*	MG973340
	*GABAr12*	MG973341

### GABA receptors

**Fig 10:**
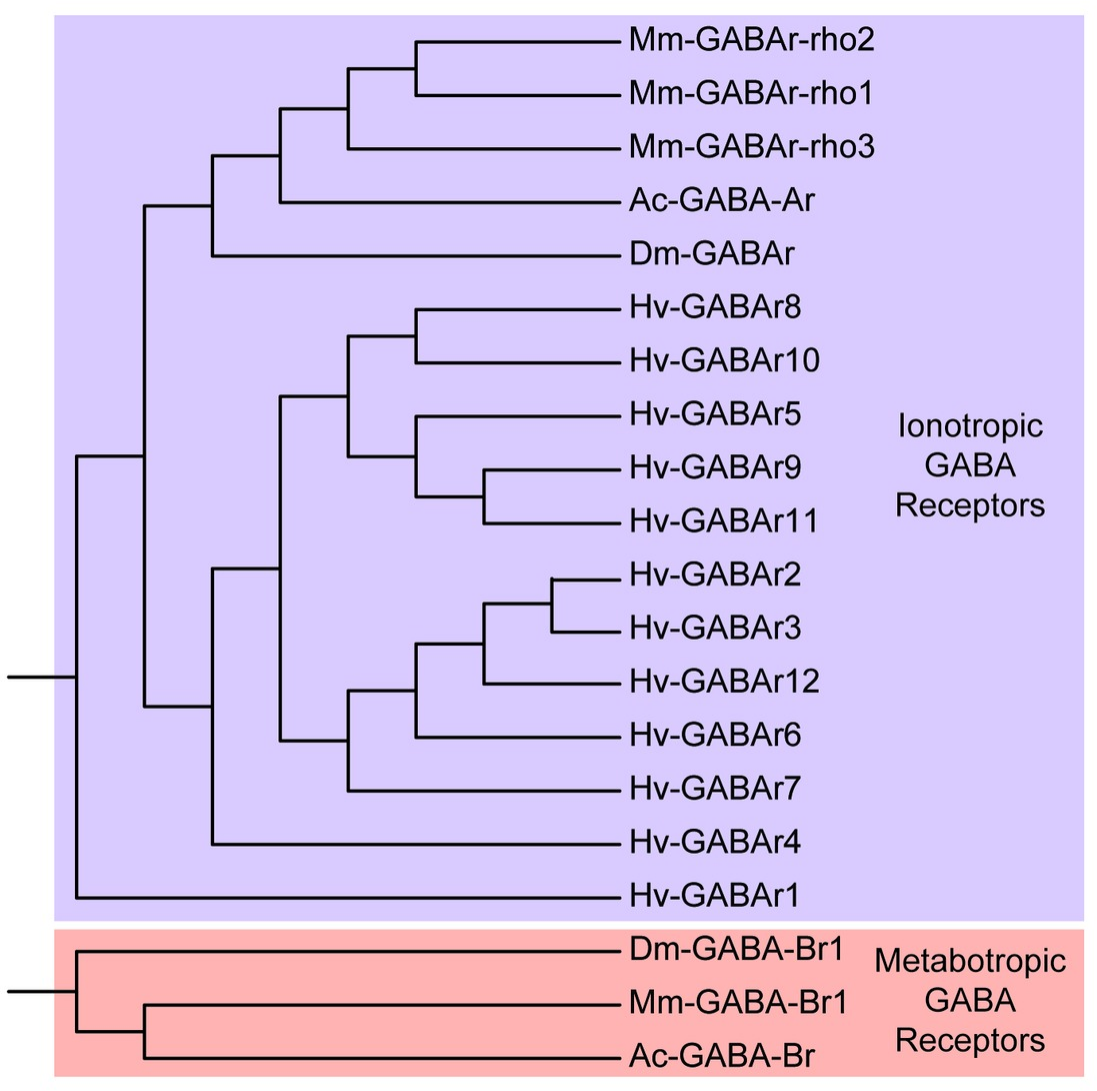
GABA receptors identified in the *H*. *verbana* CNS transcriptome. Trees were produced in the same manner as in Fig 4. *H*. *verbana* GABA receptor subtypes and accession numbers can be found in Table 5.

From the *H*. *verbana* CNS transcriptome, 12 putative GABA receptor contigs were identified (Table 5). While these sequences were identified as GABA receptors using BLAST top hits from *D*. *melanogaster*, *M*. *musculus*, and *A*. *californica* (see Methods), we also noted that they shared some sequence similarity with glycine receptors as well, which perhaps is not surprising considering both receptor families include ionotropic receptors that produce Cl^-^ currents. The identified *H*. *verbana* GABA receptors were more similar to ionotropic rather than metabotropic GABA receptors from other species (Fig 10).

### Glutamate receptors

**Fig 11:**
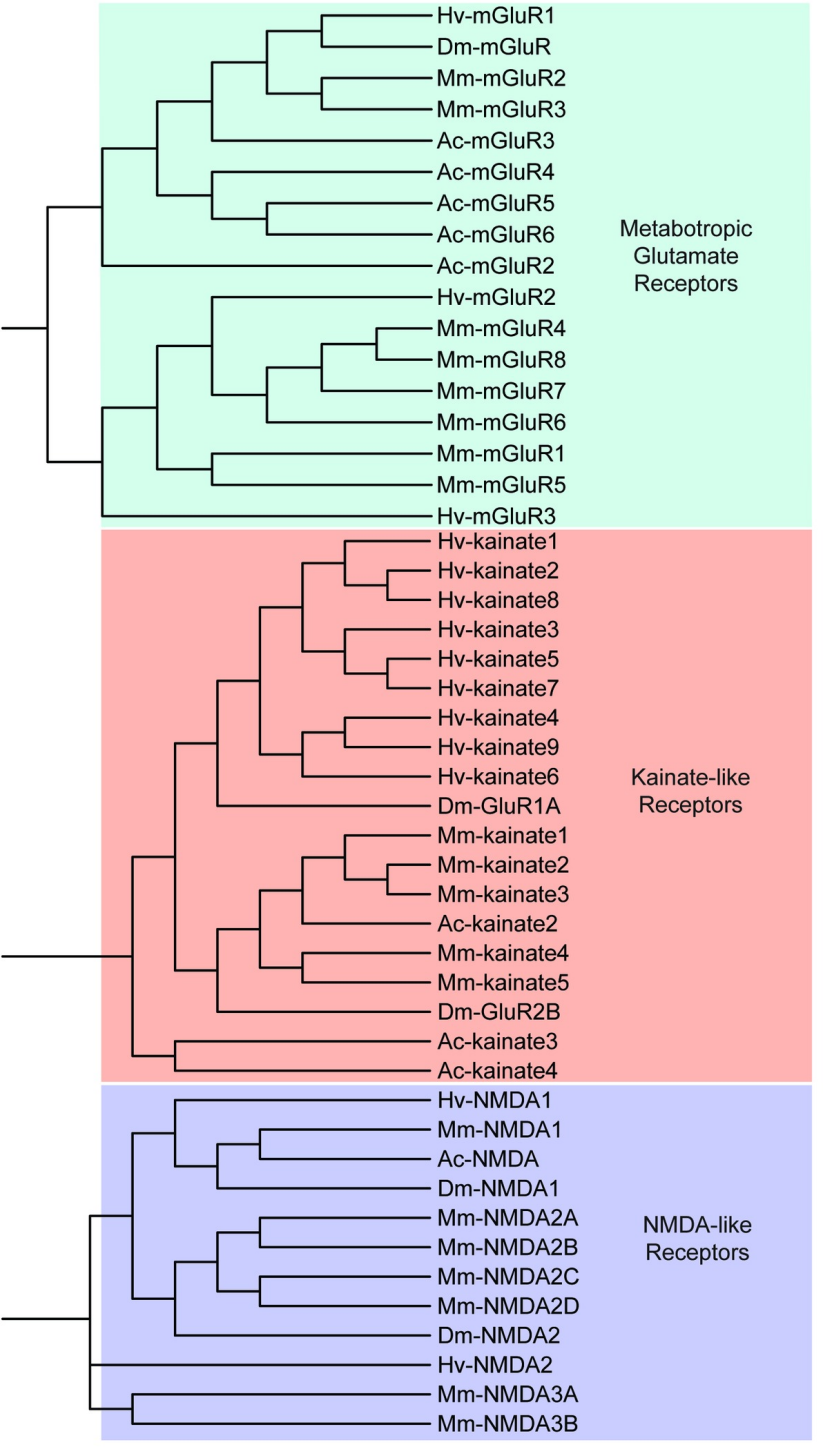
Ionotropic (kainate- and NMDA-like) and metabotropic glutamate receptors identified in the *H*. *verbana* CNS transcriptome. Trees were produced in the same manner as in Fig 4. *H*. *verbana* glutamate receptor subtypes and accession numbers can be found in Table 7.

In our search of glutamate receptors in the CNS transcriptome of *H*. *verbana* we identified three metabotropic glutamate receptor contigs and 11 ionotropic glutamate receptor contigs, of which we were confident enough to divide the ionotropic receptors into 9 “*kainate-like*” and two “*NMDA-like*” glutamate receptors (Table 7). Clustering of these sequences resulted in the major separation of metabotropic and ionotropic glutamate receptors, followed by the minor separation of ionotropic receptors into *kainate* and *NMDA* (Fig 11).

**Table 7:** Glutamate and acetylcholine receptor accession numbers from CNS transcriptome.

Receptor Family	Gene Name	*H*. *verbana acession*
**Metabotropic Glutamate Receptors**	*mGluR1*	MG973342
	*mGluR2*	MG973343
	*mGluR3*	MG973344
**Kainate-like Receptors**	*Kainate1*	MG973345
	*Kainate2*	MG973346
	*Kainate3*	MG973347
	*Kainate4*	MG973348
	*Kainate5*	MG973349
	*Kainate6*	MG973350
	*Kainate7*	MG973351
	*Kainate8*	MG973353
	*Kainate9*	MG973355
**NMDA-like Receptors**	*NMDA1*	MG973352
	*NMDA2*	MG973354
**Acetylcholine Receptors**	*AChR1*	MG973297
	*AChR2*	MG973298
	*mAChR1*	MG973313
	*mAChR2*	MG973314
	*mAChR3*	MG973315
	*mAChR4*	MG973316
	*nAChR1*	MG973299
	*nAChR2*	MG973300
	*nAChR3*	MG973301
	*nAChR4*	MG973302
	*nAChR5*	MG973303
	*nAChR6*	MG973304
	*nAChR7*	MG973305
	*nAChR8*	MG973306
	*nAChR9*	MG973307
	*nAChR10*	MG973308
	*nAChR11*	MG973309
	*nAChR12*	MG973310
	*nAChR13*	MG973311
	*nAChR14*	MG973312

### Acetylcholine receptors

**Fig 12:**
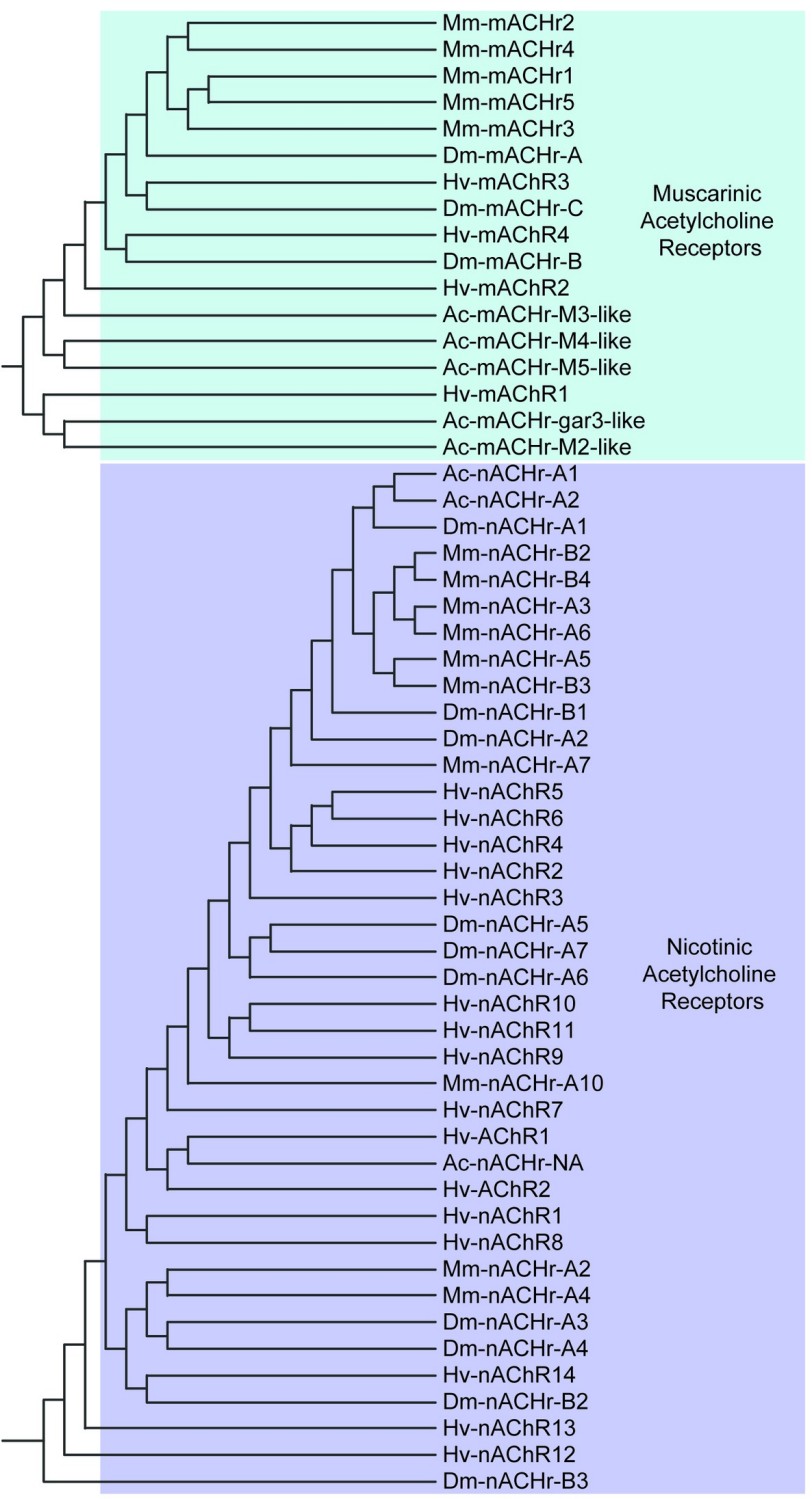
Muscarinic and nicotinic acetylcholine receptor families identified in the *H*. *verbana* CNS transcriptome. Trees were produced in the same manner as in Fig 4. *H*. *verbana* acetylcholine receptor subtypes and accession numbers can be found in Table 7.

The acetylcholine receptor content of the *H*. *verbana* CNS transcriptome includes 20 putative acetylcholine receptor contigs, of which 4 were identified as muscarinic, 14 as nicotinic, and 2 that were not identified as muscarinic or nicotinic based on ambiguity in their BLAST results (Table 7). The clustering of the acetylcholine receptors resulted in a clear distinction between the muscarinic and nicotinic acetylcholine receptors (Fig 12). Note that the two unclassified acetylcholine receptors clustered more similarly to nicotinic acetylcholine receptors than muscarinic ones.

### Transmitter-related genes

**Fig 13:**
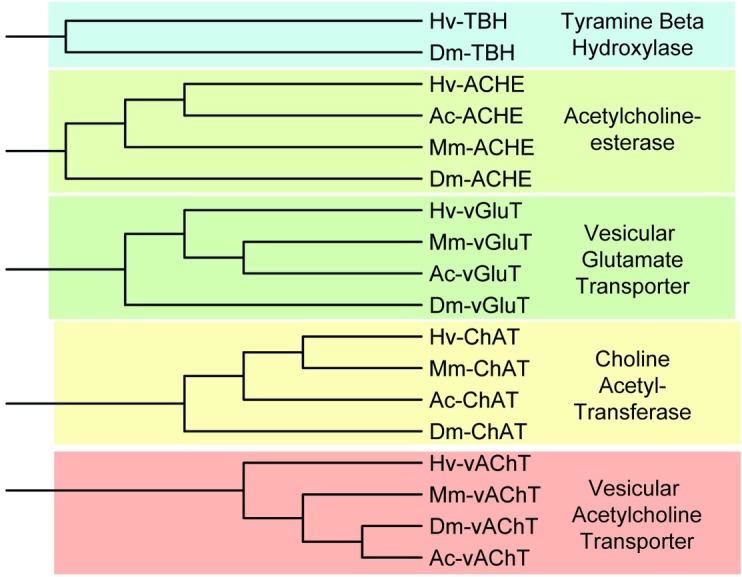
Neurotransmitter-associated enzymes and transporters identified in the *H*. *verbana* CNS transcriptome. Trees were produced in the same manner as in Fig 4. Transmitter-related subtypes and accession numbers can be found in Table 8.

Beyond ion channels and receptors, the genes that synthesize, break down, and transport neurotransmitters are important for distinguishing neuronal types throughout the nervous system. We specifically identified putative tyramine beta-hydroxylase (*TBH)*, acetylcholinesterase (*ACHE*), vesicular glutamate transporter (*vGlutT*), choline acetyltransferase (*ChAT*), and vesicular acetylcholine transporter (*vAChT*) orthologous contigs (Table 8). Clustering of the *H*. *verbana* transmitter-related sequences with the *A*. *californica*, *M*. *musculus*, and *D*. *melanogaster* corresponding sequences created five distinct clusters (Fig 13).

**Table 8:** Transmitter-related enzyme and transporter accession numbers from CNS transcriptome.

	Gene Name	*H*. *verbana accession*
**Transmitter-related**	*TBH*	MG973413
	*ACHE*	MG973414
	*vGluT*	MG973415
	*ChAT*	MG973416
	*vAChT*	MG973417

### Ion channel RNA-seq counts

**Fig 14:**
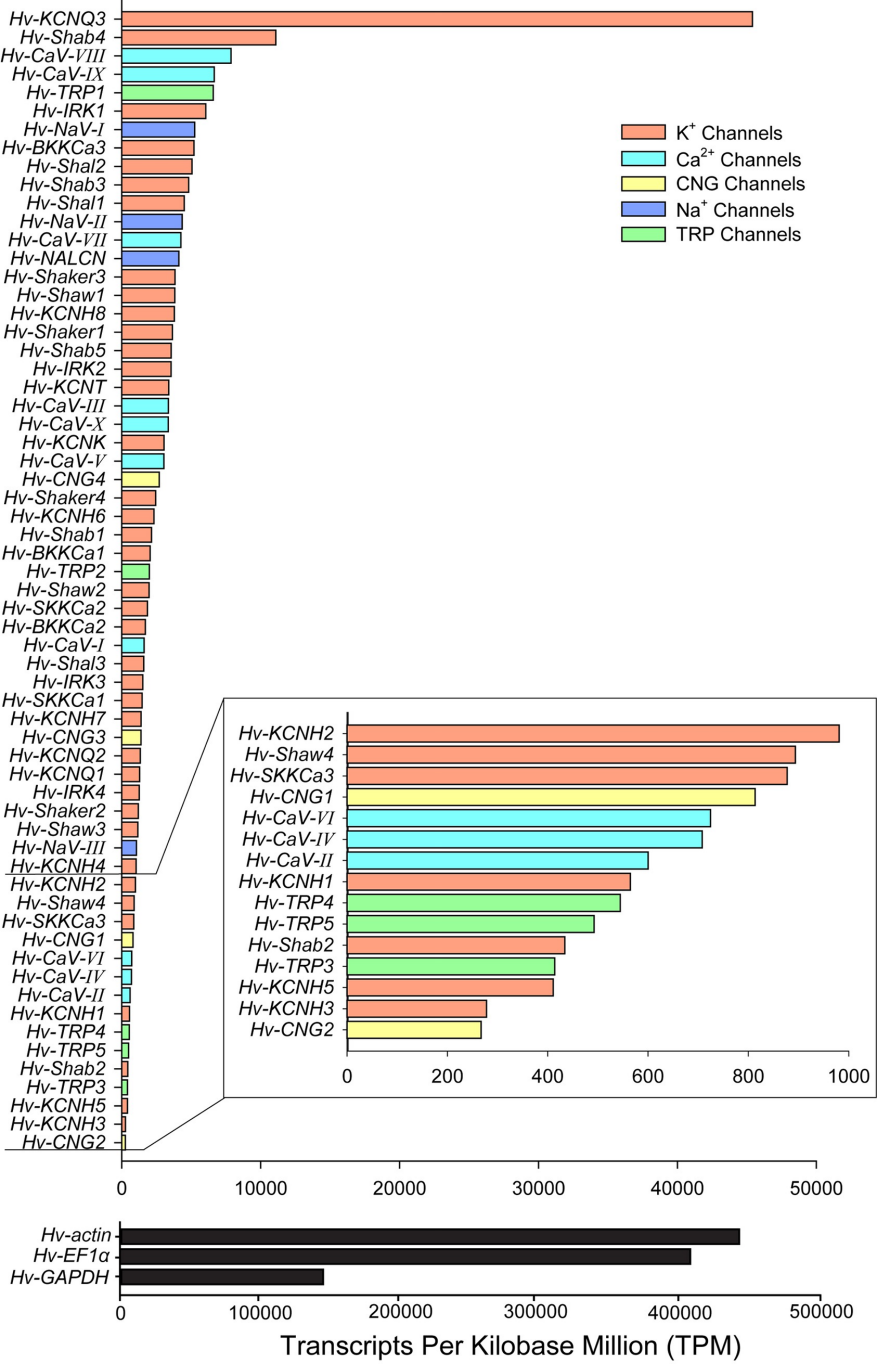
RNA-seq kallisto counts for ion channels and housekeeping genes identified in the *H*. *verbana* CNS transcriptome. Horizontal bar plot ordered based on read alignment with color coordinating to channel families. Read counts were normalized using TPM method. Inset displays zoomed in visualization of channels where TPM fell below 1000. Housekeeping genes are displayed with a scale bar that is an order of magnitude greater than that of the ion channels.

In order to gauge a rough abundance of the contribution of each ion channel contig to the CNS of *H*. *verbana*, all paired-end reads were mapped to the reference transcriptome using Kallisto [42] mapping software to generate a transcripts per kilobase million (TPM) normalized count of read alignments to each sequence (Fig 14). We want emphasize that while these RNA-seq counts represent a sample size of N = 1, these data are still useful in comparing relative transcript abundances. We also included the housekeeping genes *GAPDH* (contig DN38861), *EF1α* (contig DN32737), and *actin* (contig DN35234) to give a sense of relative channel and receptor abundance. By far, the most abundant ion channel transcript is *KCNQ3*, a voltage-gated slow delayed rectifier K^+^ channel. For Ca^2+^ channels, *CaV-VIII* and *CaV-IX* both show strong expression, while *CaV-VI*, *CaV-IV*, and *CaV-II* have relatively low expression. The Na^+^ channel family has *NaV-I* as its most abundant member, followed closely by *NaV-II* and *NALCN*. The predominant TRP channel expressed is *TRP1* with abundances greater than that of *TRP2-5*. The CNG channels overall seem to be less abundant in the CNS of the leech, with *CNG4* as its most abundant member. However, even the ion channels with a TPM < 1000 could still play an important role in neural function, particularly if they are only expressed in certain neuron types, which could explain the lower relative abundance in the whole CNS.

### Receptor RNA-seq counts

**Fig 15:**
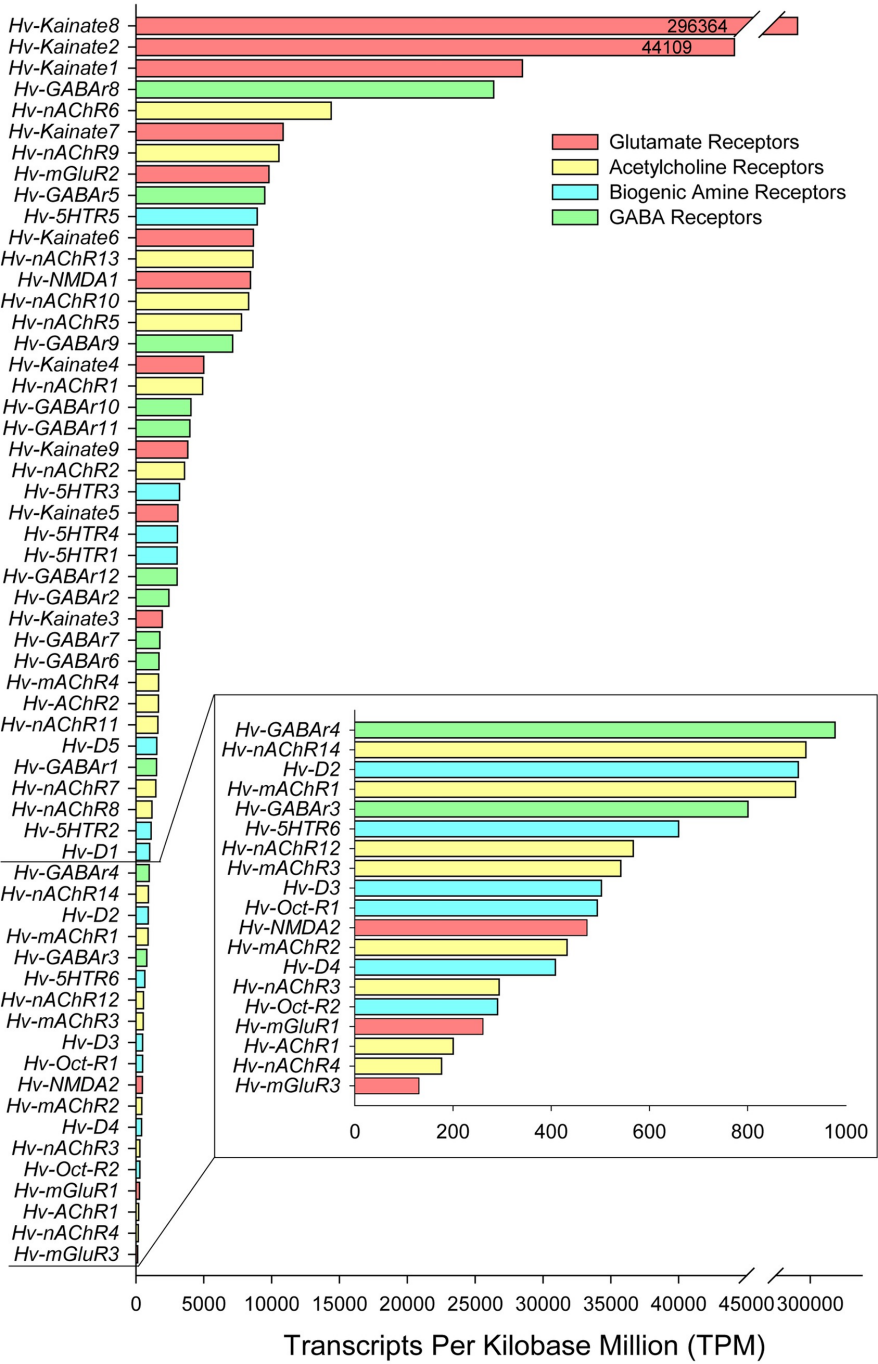
RNA-seq kallisto counts for receptor contigs identified in the *H*. *verbana* CNS transcriptome. Horizontal bar plot ordered based on read alignment, with color coordinating to receptor families. Read counts were normalized using TPM method. Inset displays zoomed-in visualization of receptors where TPM fell below 1000. Line break is indicated as two dashed lines where *Hv-Kainate8* greatly exceeded read counts compared to other receptor contigs. Absolute values of TPM normalized read counts are displayed within the bars for *Hv-Kainate8* and *Hv-Kainate2* to accentuate the noteworthy differences in abundance.

Repeating the same RNA-seq Kallisto mapping analysis for receptors yielded a greater overall abundance than that of the ion channel contigs (Fig 15). Particularly, kainate-like receptors were massively abundant in the transcriptome. The putative ionotropic glutamate receptor *Kainate-8* contig was nearly an order of magnitude greater than the next most abundant sequence, *Kainate-2*, as is noted with the line break where *Kainate-8* approaches a TPM of nearly 300,000. While kainate-like orthologs in the CNS certainly seem to be the dominant ionotropic glutamate receptor (holding the top 3 most abundant positions), *NMDA1* also generated a fairly large TPM. The metabotropic glutamate receptors have only one strongly expressed member: *mGluR2*; *mGluR1* and *mGluR3* both have some of the lowest receptor abundances out of the data set. For GABA receptor contigs, *GABAr8* is the most abundant, with *GABAr5* and *GABAr9* having strong abundance as well. The acetylcholine receptor abundance favors nicotinic acetylcholine receptors over muscarinic, with 7 nicotinic receptors having higher abundance than the highest muscarinic, *mAChR4*. The highest expressed nicotinic acetylcholine receptor contig is *nAChR6*. The two acetylcholine receptor contigs that were not given muscarinic or nicotinic designations had generally lower abundances as well. For the biogenic amine contigs, serotonin receptors dominate with expression far exceeding that of octopamine and dopamine. However, dopamine receptorcontigs still show much stronger abundance than octopamine receptors, which had some of the lowest expression levels noted.

## Discussion

This study provides the first annotated CNS transcriptome for the leech *H*. *verbana*. A previously generated nervous system transcriptome obtained from *H*. *medicinalis* provided some of the first extensive RNA-seq data for a related leech species [16], but that transcriptome used selected ganglia (G2, G10, and G19) to examine spatial differences in expression patterns, whereas the present study surveyed the entire CNS. The inventory of ion channels and receptor contigs from the CNS transcriptome is a valuable community resource for further investigations. Prior to our work, NCBI had 110 entries for *H*. *verbana* amino acid sequences, derived primarily from genes encoding functions related to the mitochondria, coagulation, and gap junctions (innexins). In the present study, we have more than doubled the total number of individually curated and annotated GenBank sequences for *H*. *verbana* through the mining of this CNS transcriptome for transcripts related to neural function.

An extensive characterization of Na^+^, K^+^, and Ca^2+^ currents in the leech CNS has previously been examined using voltage-clamp measurements, ion replacement, and pharmacological blockers to understand the role that these ionic currents play in cultured leech Retzius (R), anterior pagoda (AP) and sensory neurons, attributing different membrane properties to distinct ion channel complements [49]. In the pressure-sensitive mechanosensory (P) neurons, it was shown that the probabilities of K^+^-channel open and closed states could be modulated by different phosphorylation events mediated by a *5HTR* subtype [50]. The fast-transient A-type potassium current also increased with the age of cultured AP neurons, while other potassium currents remained unchanged [51]. Through patch clamp experiments, the potassium leak channel *KCNK* has been shown to be affected by axotomy of AP neurons through patch clamp experiments, which showed that the number, but not the properties, of *KCNK* channels was changed following the loss of an axon [52]. A persistent cesium-sensitive inward current, potentially carried by *IRK*, has also been characterized in cultured R cells with voltage-clamp measurements during pharmacological blockade [53]. The dynamics of the Na^+^-activated K^+^ channel KCNT have been examined in leech P cells where membrane resistance was strongly influenced by changes in outwardly directed membrane current with changing intracellular Na^+^ concentration [54].

While our analysis identified ten sequences in the Ca^2+^ channel family, in this study we did not functionally classify these channels into further subcategories, such as L-, N-, R-, T-, or P/Q-type. Such one-to-one classification between mammals and invertebrates or non-mammlaian vertebrates can be problematic, and is better done thorough a combination of firsthand verified full-length sequence information combined with pharmacological profiling of the resulting currents (e.g. [55,56]). Other investigations into the Ca^2+^ channel content of the leech CNS have suggested that the vast majority of leech neurons contain voltage-gated Ca^2+^ channels similar to L-type channels found in vertebrates [57], as well as showing heart neurons with Ca^2+^ dynamics that resemble T-type Ca^2+^ channels. L-type Ca^2+^ channels have also been implicated in the somatic release of serotonin in cultured R cells [58]. In *H*. *medicinalis*, four putative Na^+^ alpha subunits have been previously identified that were sequenced through subcloning, and RT-PCR revealed their differential expression across cell types of the nervous system [59]. Our analysis found three putative *H*. *verbana* Na^+^ channels which most closely matched the 1, 2, and 4 isoforms of *H*. *medicinalis*. Sodium-channel blocking tetrodotoxin (TTX) sensitivity in leech Retzius neurons displayed altered responses to frequency stimulations, suggesting a role of TTX-sensitive sodium channels in sensitization and habituation [60].

The non-specific cation TRP channels are thought to be the primary channel responsible for nociceptive, temperature, and pressure sensations. Multiple responses to nociceptive stimuli in neurons residing within the leech segmental ganglia have been reported, with cells responding to capsaicin, mechanical stimuli, temperature perturbations, and pH changes [61]. The five *H*. *verbana* TRP channels identified in this study were similar to vanilloid (*TRPV*) or ankyrin (*TRPA*) as compared to other types, but classifying these five channels further proved difficult. Evidence supports the presence of capsaicin-sensitive TRPV channels from capsaicin responses in nociceptive neurons being reduced in the presence of a selective TRPV1 antagonist [62]. Endocannabinoid pathways have also been shown in the leech nervous system to be reliant on TRPV channels [63].

Both ionotropic and metabotropic glutamate receptors have been studied for their role in the CNS of the leech. Metabotropic glutamate receptors in the leech, of which we identified three putative sequences in this study, have been found to increase intracellular Ca^2+^ by drawing on intracellular Ca^2+^ stores when mGluR-selective agonists were applied [64]. Two unpublished ionotropic glutamate receptors have been previously identified, simply described as glutamate receptor 1 and 2 (Accession Numbers ARJ36889.1 and AGL96589.1), which correspond to sequences we have identified as *Hv-Kainate1* and *Hv-kainate2*, respectively. Ionotropic glutamate receptors were identified throughout the neuropil and neural somata within ganglia comprising the ventral nerve cord using immunostaining procedures [65]. Our present study identified 9 kainate-like ionotropic glutamate receptors. These receptors most resembled kainate-like and not AMPA-like receptor orthologs; however, these pharmacological designations cannot be determined directly from sequence data, and therefore such receptors can only be identified as a distinct subtype family from the putative NMDA-like receptors. While the vast majority of ionotropic glutamate receptors were kainate-like, we also identified two NMDA-like receptors. NMDA receptors have been implicated as a requirement for the production of long-term potentiation (LTP) in the leech nervous system [66]. One partial NMDA-receptor has been previously identified (Accession No. ACE95895.1), which most closely resembles *Hv-NMDA1* from *H*. *verbana*.

In previous studies, autoradiographically-labeled GABAergic neurons were observed throughout the ventral nerve cord of the leech, revealing approximately thirty GABAergic neurons per each segmental ganglion in bilaterally paired or unpaired configurations [67]. It has been shown that GABAergic synaptic responses in the leech can have either excitatory or inhibitory effects, inducing hyperpolarization in pressure-sensitive cells while depolarizing nociceptive cells, mediated in part by Cl^-^ homeostasis [68].

Dopamine and serotonin have been extensively studied in connection with many behaviors in the leech. Dopamine has been found to activate fictive crawling in locomotor CPG networks [69] while also inhibiting swimming behavior [70]. Fictive swimming behaviors can also be inhibited through application of both serotonin and octopamine to the brain [71] or promoted when administered separately to the entire nervous system [72]. Serotonin has further been implicated in increasing force production in the body wall muscles associated with locomotion and feeding behaviors [73]. In the recently identified stomatogastric nervous system (STN) of *H*. *verbana*, serotonin-immunopositive somata were identified in the stomatogastric ganglia (STGs), while no dopaminergic somata were found to be present [74], further strengthening the association of serotonin with feeding behavior.

Our analysis of acetylcholine receptors from the leech CNS transcriptome yielded 20 different putative receptor contigs, with 14 being nicotinic-like, 4 being muscarinic-like, and two acetylcholine receptors that were unclassified into a subfamily. In the leech, acetylcholine and the muscarinic agonist carbachol have been shown to inhibit heart interneurons and motor neurons [75]. Acetylcholine application has also been shown to increase both the frequency and total number of action potentials in leech pressure-sensitive cells [76]. Removal of the axon from the leech anterior pagoda neuron has been shown to reduce the density of acetylcholine receptors in that neuron, leading to reduced sensitivity to acetylcholine [77]. Acetylcholine has also been shown to mediate Ca^2+^ increases in glial cells of the neuropil through the action of nicotinic acetylcholine receptors [78].

Without the enzymes and transporters responsible for synthesizing and concentrating neurotransmitters, neuronal communication as we know it would not be possible. The enzyme responsible for converting tyramine to octopamine is tyramine beta-hydroxylase (TBH), which has been shown through antibody labeling to be associated with identified octopaminergic neurons in the cephalic and terminal ganglia of the leech [48]. The acetylcholine synthesizing enzyme choline acetyltransferase (ChAT) has been detected in excitatory motor neurons of the leech CNS, but not in the Retzius cells or mechanosensory cells [79]. Acetylcholinesterase (ACHE) activity has been found throughout the leech CNS, as well as in the blood, with most ACHE-positive neurons being cholinergic motoneurons [80]. The vesicular acetylcholine transporter (vAChT) and vesicular glutamate transporter (vGluT) were also identified in this study; to the best of our knowledge, this is the first identification of these sequences in the medicinal leech.

## Conclusions

This transcriptomic study lays a foundation for further molecular analyses in the leech preparation that has been a stalwart contributor to our understanding of the fundamentals of nervous system function and behavior. The 126 ion channels, receptors, transporters, and enzyme contigs individually described in this study, as well as the entire annotated CNS transcriptome, provide a strong reference for not only *H*. *verbana*, but future comparative analyses across the nervous systems of related species [34]. These data stand as a substantial foundation for future work with more focused bioinformatics, sequence assembly, and supplementation with further sequencing approaches to curate fully genes of interest related to neural function. With high-quality transcripts readily available, the additional incorporation of qRT-PCR, siRNA, overexpression [81], and RNA-seq approaches promise to lower the hurdles towards addressing some of the outstanding issues remaining to be addressed in the neurosciences.

## Supporting information

S1 File Gene ontology annotation for *H*. *verbana* CNS transcriptome. (GZ)
